# Self-enriching nanozyme with photothermal-cascade amplification for tumor microenvironment-responsive synergistic therapy and enhanced photoacoustic imaging

**DOI:** 10.1016/j.mtbio.2025.102230

**Published:** 2025-08-23

**Authors:** Xi Zhu, Yang Zhang, Yufei He, Li Li, Xiaofei Luo, Ran Zhao, Xiaoying Yan, Ceshi Chen

**Affiliations:** a Yunnan Key Laboratory of Breast Cancer Precision Medicine, Institute of Biomedical Engineering, Kunming Medical University, Kunming, 650500, Yunnan, China; b Department of Ultrasound, Shandong Provincial Hospital Affiliated to Shandong First Medical University, Jinan, 250021, Shandong, China; c Faculty of Basic Medical Sciences, Kunming Medical University, Kunming, 650500, Yunnan, China; d Gene Regulation and Diseases Lab, College of Life Science and Technology, College of Biomedicine and Health, Huazhong Agricultural University, Wuhan, 430070, Hubei, China; e Department of Mechanical and Vehicle Engineering, Changsha University of Science and Technology, Changsha, 410114, Hunan, China; f Yunnan Key Laboratory of Breast Cancer Precision Medicine, Yunnan Cancer Hospital, The Third Affiliated Hospital of Kunming Medical University, Yunnan Hospital of Peking University Cancer Hospital, Kunming, 650118, Yunnan, China

## Abstract

Achieving precise intratumoral accumulation and coordinated activation remains a major challenge in nanomedicine. Photothermal therapy (PTT) provides spatiotemporal control, yet its efficacy is hindered by heterogeneous distribution of PTT agents and limited synergy with other modalities. Here, we develop a dual-activation nanoplatform (IrO_x_-P) that integrates exogenous photothermal stimulation with endogenous tumor microenvironment (TME)-responsive catalysis for synergistic chemodynamic therapy (CDT) and ferroptosis induction. The IrO_x_ core exhibits robust peroxidase- and catalase-like activities, enabling Ir^3+^/Ir^4+^ redox cycling for glutathione depletion, hydroxyl radical generation and O_2_ production. Surface conjugation of P-selectin targeting peptides directs selective binding to activated platelets. Upon mild PTT, vascular injury induces platelet activation, triggering secondary self-enrichment of IrO_x_-P at tumor sites and amplifying catalytic activity. This cascade enhances CDT/ferroptosis efficacy while enabling O_2_-augmented photoacoustic imaging for real-time monitoring. The strategy establishes a self-recruitment nanotheranostic paradigm that couples PTT-induced biological effects with catalytic nanomedicine, offering a versatile approach for precision cancer therapy.

## Highlights

• Photothermal-cascaded nanozyme catalysis amplifies chemodynamic and ferroptosis for synergistic therapy.
• Dual-responsive activation strategy achieves integrated spatiotemporal therapeutic coordination.
• P-selectin-guided platelet recruitment mechanism enhances tumor-specific nanoparticle enrichment.
• H_2_O_2_-triggered O_2_ bubble generation boosts photoacoustic imaging via cavitation-enhanced contrast.

## Graphical abstract

**Figure:**
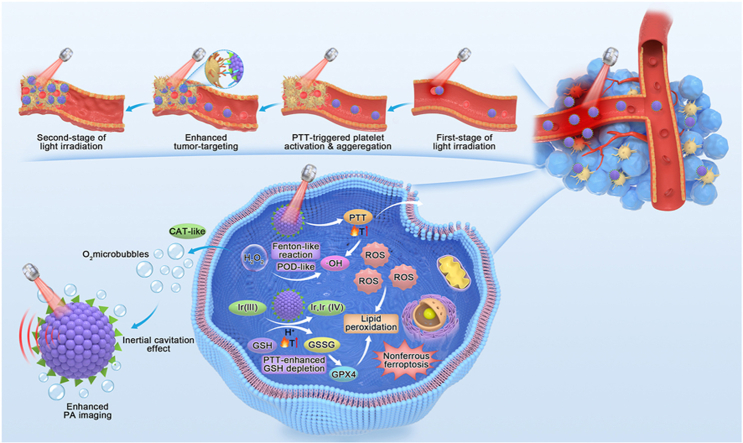

## 1 Introduction

Cancer therapy continues to face substantial challenges in achieving complete tumor eradication and preventing recurrence. Conventional modalities such as chemotherapy and radiotherapy have markedly improved patient survival, yet their therapeutic efficacy is often constrained by systemic toxicity, non-specific biodistribution, and the emergence of drug resistance. PTT, which employs photosensitive agents to convert near-infrared (NIR) light into localized heat, has emerged as a minimally invasive approach capable of selectively ablating tumor tissues with high spatial precision [1,2]. PTT offers several advantages, including controllable treatment depth, reduced systemic side effects, and compatibility with other therapeutic strategies [3,4]. Nevertheless, its long-term efficacy is frequently compromised by insufficient nanoparticle accumulation at the tumor site, heterogeneous heat distribution, and the risk of residual tumor cell survival leading to recurrence [5]. These limitations underscore the need for rationally designed combination regimens that can integrate PTT with complementary therapeutic modalities, enabling spatiotemporally coordinated, multi-mechanistic tumor eradication and concurrent diagnostic monitoring.

To address these challenges, increasing attention has been directed toward integrating PTT with catalytic nanomedicine, particularly tumor nanocatalysis therapy. This strategy employs engineered nanozymes that mimic the electron transfer processes of natural redox enzymes to generate cytotoxic reactive oxygen species (ROS) within the tumor microenvironment [6,7]. By incorporating metal-based nanozymes with peroxidase (POD)- or oxidase-like activity, these systems catalyze the in situ production of ROS within tumors, thereby inducing both CDT and ferroptosis [[8], [9], [10]]. In CDT, transition metal ions (typically Fe^2+^ or Cu^+^) catalyze Fenton or Fenton-like reactions, converting tumor-overexpressed H_2_O_2_ into highly cytotoxic hydroxyl radical (·OH) [[11], [12], [13]]. Ferroptosis, an iron-dependent form of regulated cell death, involves similar biochemical pathways in which excessive LPO accumulate and overwhelm cellular antioxidant defenses, particularly the glutathione glutathione (GSH)/glutathione peroxidase 4 (GPX4) axis [14]. Consequently, strategies that can concurrently enhance intracellular ROS production while depleting GSH to impair antioxidant defenses have emerged as a prominent research focus.

According to the Arrhenius equation, the rate of catalytic reactions increases exponentially with temperature [15]. Recent studies have demonstrated that PTT can accelerate redox and Fenton reactions, thereby amplifying the efficacy of CDT and ferroptosis [9,10,16,17]. However, realizing precise spatiotemporal coordination, where catalytic activation and photothermal stimulation occur synchronously within the tumor microenvironment, remains a key challenge. Such coordination depends critically on achieving high and controllable intratumoral accumulation of nanoagents. This goal is often compromised by tumor heterogeneity and the inherent limitations of conventional targeting approaches, which typically rely on single-ligand recognition (e.g., antibodies) of tumor-associated antigens [[9], [18], [19], [20]]. Exploiting the multifaceted physical, chemical, and biological effects induced by PTT offers an alternative route to on-demand targeting and enrichment. For example, photothermal-induced thermal gradients can drive self-propulsion of nanomotors [21], while localized vascular injury and platelet activation triggered by mild hyperthermia can be leveraged for platelet membrane- or platelet particle-mediated targeting [22,23]. Nonetheless, these strategies face persistent limitations, including suboptimal in vivo stability, high production costs, and incomplete integration with TME-responsive catalytic modalities such as CDT and ferroptosis. Addressing these gaps requires the development of platforms capable of coupling precise photothermal activation with robust catalytic functionality in a unified therapeutic framework.

**Fig. 1:**
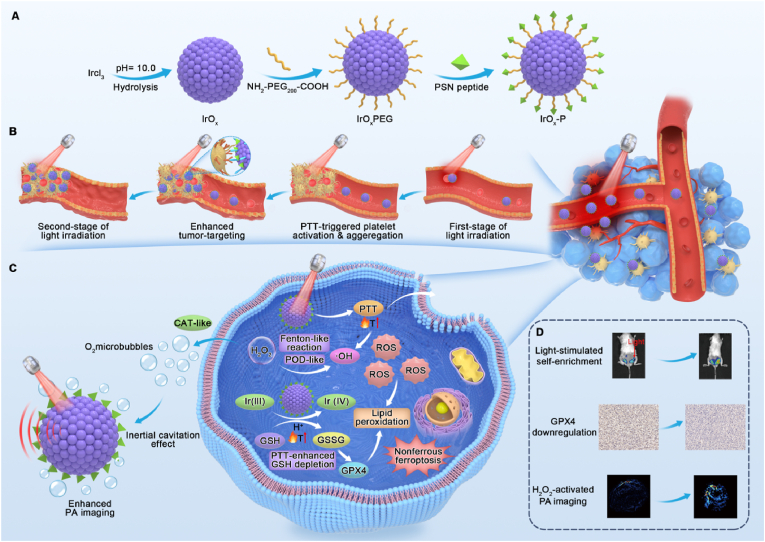
**Integrated design and multimodal theranostic mechanism of IrO_x_-P nanoparticles. (A)** Synthesis and functionalization process of IrO_x_-P nanoparticles. IrO_x_ nanoparticles were synthesized via a hydrolysis method, followed by PEG modification and conjugation with PSN to form IrO_x_-P. The resulting nanoparticles exhibit excellent photothermal properties, enzymatic activities, and tumor-targeting capabilities. **(B)** Photothermal irradiation induces vascular endothelial damage at the tumor site, which triggers the recruitment of activated platelets. These activated platelets highly express P-selectin, enabling specific binding to the PSN conjugated on the surface of IrO_x_-P nanoparticles. This interaction mediates secondary enrichment of IrO_x_-P at the irradiated site, thereby enhancing local accumulation and subsequent therapeutic efficacy. **(C)** Schematic diagram illustrating the dual-responsive catalytic mechanism of IrO_x_-P for synergistic CDT/ferroptosis and PA imaging. **(D)** Representative images of light-stimulated self-enrichment, GPX4 downregulation and H_2_O_2_-acitivated PA imaging.

Here, we designed a novel activated platelet-targeted iridium oxide (IrO_x_-P) nanoparticles that integrates PTT with ROS-mediated CDT and ferroptosis in a synergistic, spatiotemporally coordinated manner. The IrO_x_-P nanoparticles consist of an IrO_x_ core, which provides both photothermal and catalysis functions via Ir^3+^/Ir^4+^ redox cycling, and a surface conjugation of P-selectin-targeting peptides (PSN), yielding the final IrO_x_-P nanocomposite (Fig. 1A). This dual-responsive system combines exogenous photothermal activation with endogenous TME-specific catalytic activation. Following systemic administration, IrO_x_-P nanoparticles initially accumulate in tumors through the enhanced permeability and retention (EPR) effect. Upon mild photothermal irradiation, localized vascular injury induces the activation of P-selectin-expressing platelets [[22], [23], [24]], which selectively bind PSN, thereby promoting secondary nanoparticle recruitment to the irradiated tumor site (Fig. 1B). This self-reinforcing enrichment amplifies therapeutic payload delivery. The first therapeutic phase arises from PTT-induced hyperthermia, which directly induces tumor cell apoptosis. Subsequently, under the acidic and H_2_O_2_-rich TME, PTT-enhanced IrO_x_ catalytic activity depletes GSH and generates highly reactive ·OH through POD-like activity, thereby promoting GPX4 downregulation and LPO accumulation [25,26]. This cascade effect potentiates CDT and ferroptosis, yielding a synergistic tumoricidal response. In addition to its therapeutic functions, IrO_x_-P serves as a photoacoustic (PA) imaging agent. In presence of H_2_O_2_, its catalase (CAT)-like activity produces O_2_ microbubbles, which enhance PA signals via non-inertial cavitation effects (Fig. 1C and D) [27]. Thus, IrO_x_-P enables multimodal therapy combined with real-time imaging guidance. By coupling photothermal activation with TME-responsive catalysis, this platform achieves precise spatiotemporal coordination, offering a robust and versatile nanotheranostic strategy with substantial potential for clinical translation in precision oncology.

## 2 Results and discussion

### 2.1 Synthesis and characterization of IrO_x_-P

**Fig. 2:**
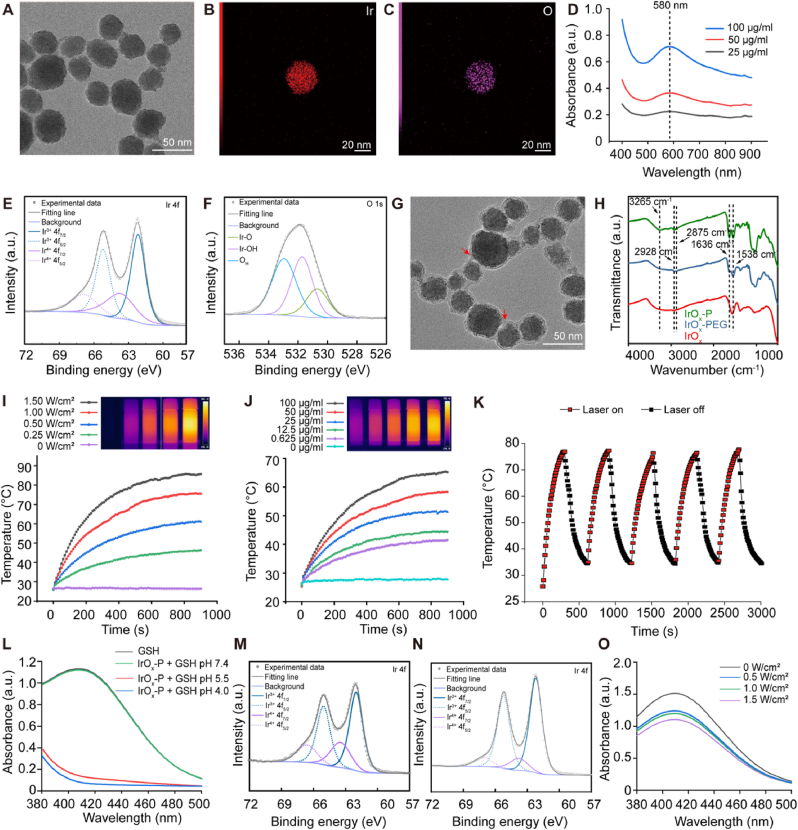
**Characterization of IrO_x_-P. (A)** TEM image of IrO_x_ and **(B and C)** the corresponding elemental Ir and O mapping images. **(D)** Absorption spectra of IrO_x_ nanoparticles at different concentrations. **(E)** Ir 4f and **(F)** O 1s XPS spectrum of the IrO_x_ nanoparticles. **(G)** TEM image of IrO_x_-P. **(H)** FTIR spectra of IrO_x_-P. Absorption peaks at 1636 cm^−1^ and 1538 cm^−1^ are attributed to the amide I and amide II bands, respectively. Peaks at 2928 cm^−1^ and 2875 cm^−1^ are attributed to C-H stretching vibrations, while the peak at 3265 cm^−1^ represents the N-H stretching vibration. **(I)** Photothermal heating curves of IrO_x_-P (50 μg/mL) under 808 nm laser irradiation with different power density. **(J)** Photothermal heating curves of IrO_x_-P with different concentration (808 nm, 0.5 W/cm^2^). **(K)** Photostability curves of IrO_x_-P nanoparticles (50 μg/ml) within five on-off cycles (808 nm, 1.5 W/cm^2^). **(L)** GSH depletion by IrO_x_-P detected by DTNB. Ir 4f XPS spectrum of IrO_x_ nanoparticles before **(M)** and after reaction with GSH **(N). (O)** GSH depletion of IrO_x_-P with laser irradiation detected by DTNB. Data are presented as mean ± s.d.

First, IrO_x_ nanoparticles were synthesized via a one-step thermal hydrolysis [28] of dissolved Ircl_3_ under alkaline conditions at 100 °C with sodium citrate serving as a stabilizing agent (Fig. 1A). This citrate-mediated approach is critical for achieving colloidal stability, as citrate anions provide both electrostatic repulsion and steric hindrance-a strategy widely employed for noble metal nanoparticles [29,30]. The reaction mechanism involved OH^−^-assisted hydrolysis of Ir precursors, with real-time pH monitoring being essential to prevent aggregation caused by electrostatic attraction between OH^−^ and H^+^ at low pH value [31,32]. As shown in Fig. S1, when pH monitoring and adjustment were not performed during the reaction, the resulting IrO_x_ did not exhibit a spherical structure but instead formed an interconnected network. In contrast, when pH was monitored every half hour and adjusted to 10, the generated IrO_x_ nanoparticles were well-dispersed spherical particles with an average diameter of 31.7 ± 4.5 nm (Fig. 2A). The characteristic elemental mapping of IrO_x_ nanoparticles indicated the uniform distribution of Ir and O (Fig. 2B and C). The IrO_x_ nanoparticles exhibited broad absorption in the near-infrared region, with a characteristic absorption peak around 590 nm, confirming the successful formation of IrO_x_ [28]. Moreover, the absorption intensity increased as the concentration increased (Fig. 2D). X-ray photoelectron spectroscopy (XPS) was applied to detect the chemical state of IrO_x_. The Ir 4f exhibited two binding peaks at ∼63.73 eV (Ir 4f_7/2_) and ∼66.84 eV (Ir 4f_5/2_), and peaks at ∼62.16 eV (Ir 4f_7/2_) and ∼65.19 eV (Ir 4f_5/2_), which could be assigned to the Ir^4+^ and Ir^3+^ (Fig. 2E). The peaks at ∼530.69 eV and ∼531.72 eV indicated the formation of Ir-O and Ir-OH (Fig. 2F).

To functionalize the IrO_x_ nanoparticles with photothermal-induced self-enrichment, IrO_x_ nanoparticles were modified with PEG-NH_2_ and then conjugated with the PSN via an amidation reaction, yielding IrO_x_-P (Fig. 1G). The elemental mapping revealed the uniform distribution of N elements in IrO_x_-P in addition to Ir and O (Fig. S2A). X-ray powder diffraction (XRD) analysis of IrO_x_-P revealed a broad diffraction peak characteristic of nanoscale crystallites, which arose from low limit of XRD towards small granules (Fig. S2B) [28]. Distinct lattice fringes corresponding to IrO_2_ were clearly resolved in high-resolution TEM images, and the selected-area electron diffraction (SAED) patterns further confirmed the polycrystalline nature of the nanoparticles (Fig. S2C). After conjugation with PSN, the hydrodynamic size of the nanoparticles increased from ∼34.6 nm to ∼49.7 nm (Fig. S3A), while the ς potential also rose from ∼-31.6 mV to ∼ -15.6 mV. Moreover, Fourier transform infrared spectra of IrO_x_-P showed featured typical N-H stretching vibrations at 3265 cm^−1^, along with enhanced peaks at 1636 cm^−1^ and 1538 cm^−1^ for amide I and II (Fig. 2H). These results collectively confirmed the successful conjugation of the PSN. The as-prepared IrO_x_-P nanoparticles showed great dispersibility and stability in both aqueous solutions and physiological environments (Fig. S3B). The conjugation efficiency of PSN peptides on IrO_x_ nanoparticles was quantified by measuring the characteristic absorbance of tryptophan at 280 nm in the supernatant after reaction (Fig. S4). Based on this analysis, the coupling efficiency of PSN on IrO_x_ nanoparticles was determined to be 7.82 % (Table S1).

### 2.2 TME activated and photothermal-enhanced GSH depletion of IrO_x_-P

We systematically investigated the photothermal performance of IrO_x_-P nanoparticles under 808 nm laser irradiation. A power density-dependent and a concentration-dependent photothermal effect were observed after irradiated with an 808 nm laser (Fig. 2I and J). The nanoparticles maintained photothermal efficiency during five heating-cooling cycles (Fig. 2K). Furthermore, the photothermal conversion efficiency was calculated to be approximately 52.6 % (Figure S5A and B, Table S2), which was higher than most recently reported inorganic photothermal-ferroptosis agents [9,10,17,33]. Even under low power density irradiation (0.5 W/cm^2^), the temperature of the IrO_x_-P solution (50 μg/mL) could rise to approximately 50 °C within 250 s. These results demonstrate that IrO_x_-P possesses excellent photothermal conversion efficiency and stability, ensuring the potential for effective tumor ablation and preventing damage to surrounding normal tissues by using a relatively lower power density.

We next assessed the GSH-depleting capacity of IrO_x_-P using the 5,5′-dithiobis (2-nitrobenzoic acid) (DTNB) assay, where residual GSH reacts with DTNB to generate 2-nitro-5-thiobenzoate (TNB^2−^), exhibiting a characteristic absorption maximum at 412 nm [34]. IrO_x_-P exhibited markedly higher GSH consumption under acidic conditions than at neutral pH (Fig. 2L, Fig. S6A), consistent with tumor-selective activation in the acidic TME while remaining inactive in normal tissues. This GSH depletion was accompanied by an increase in the proportion of Ir^3+^ from 68.62 % to 85.24 % after 60 min co-incubation with GSH (Fig. 2M and N). Temperature is a critical factor influencing the rate of redox reactions [35,36], suggesting that photothermal stimulation could further potentiate this activity. Indeed, quantitative analysis revealed that laser irradiation significantly enhanced the GSH-depleting capacity of IrO_x_-P in a power density-dependent manner (Fig. 2O, Fig. S6B).

### 2.3 TME activated and photothermal-enhanced ROS production of IrO_x_-P

**Fig. 3:**
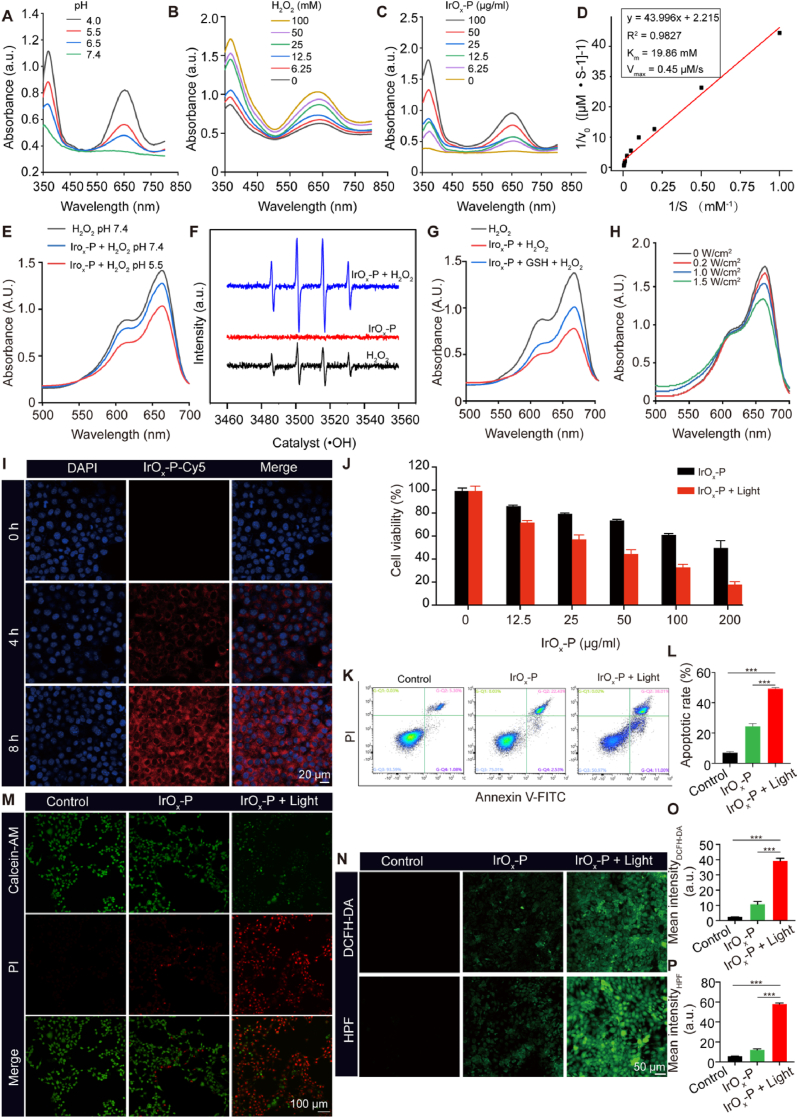
**TME-activated and PTT-enhanced ·OH production. (A)** POD-like activity of IrO_x_-P nanoparticles with different pH values detected by the absorbance of TMB oxidation. POD-like activity of IrO_x_-P nanoparticles assessed using TMB under different H_2_O_2_**(B)** and IrO_x_-P **(C)** concentrations. **(D)** Lineweaver–Burk plot for the POD-like catalytic activity of IrO_x_ nanoparticles. The initial reaction rates (v_0_) were calculated at varying H_2_O_2_ concentrations ([S]) under constant TMB concentration. The double reciprocal plot (1/v_0_ versus 1/[S]) was used to determine the Michaelis–Menten constant (K_m_) and maximum reaction velocity (V_max_). **(E)** MB degradation by IrO_x_-P-mediated ·OH production with different pH values. **(F)** ESR spectra of ·OH production by IrO_x_-P in the presence/absence of H_2_O_2_. **(G)** MB degradation by IrO_x_-P-mediated ·OH production with or without GSH pre-treatment. **(H)** MB degradation by IrO_x_-P-mediated ·OH production under 808 nm laser irradiation with different power density. **(I)** Cellular uptake of Cy5 labeled IrO_x_-P nanoparticles in 4T1 cells at different timepoints. **(J)** The relative cell viability of 4T1 cells incubated with different concentrations of IrO_x_-P with or without 808 nm laser irradiation. **(K)** Annexin V-FITC/PI-based flow cytometry analysis of 4T1 cells following different treatments. **(L)** Quantitative analysis of apoptotic cell populations (n = 3). **(M)** Fluorescence images of 4T1 cells by Calcein-AM/PI double staining after incubation with IrO_x_-P nanoparticles with or without 808 nm laser irradiation. **(N)** Fluorescence images of 4T1 cells stained with DCFH-DA or HPF probe after incubation with IrO_x_-P nanoparticles with or without 808 nm laser irradiation. Quantitative analysis of intracellular ROS **(O)** and ·OH **(P)** generation in 4T1 cells (n = 3)**.** The parameters for cell irradiation: 0.45 W/cm^2^ for 10 min. Data are presented as mean ± s.d, ∗∗∗P < 0.001.

We systematically characterized the POD-like activity of IrO_x_-P through kinetic assays and free radical analyses. The POD-like activity was quantified using TMB oxidation, where catalytic conversion of H_2_O_2_ generated a characteristic blue chromogen (*λ*_max_ = 652 nm) [28]. As shown in Fig. 3A, IrO_x_-P exhibited pH-dependent activity with optimal performance in acidic microenvironment. The POD-like activity increased with the concentration of H_2_O_2_ and IrO_x_-P (Fig. 3B and C). The POD-like catalytic activity of IrO_x_-P followed typical Michaelis-Menten kinetics at pH 4.0, yielding a K_m_ value of 19.86 mM (Fig. 3D), which is consistent with recently reported values for IrO_x_-based nanozymes [28]. For ·OH detection, methylene blue (MB) degradation assays (*λ*_max_ = 665 nm) revealed a 1.5-fold increase in reaction rate at pH 5.5 versus pH 7.4 (Fig. 3E, Fig. S7A). The electron spin resonance (ESR) spectroscopy with 5,5-dimethyl-1-pyrroline N-oxide (DMPO) trapping confirmed ·OH generation, showing the distinctive 1:2:2:1quartet signal in IrO_x_-P/H_2_O_2_ systems (Fig. 3F). Pre-incubation with GSH elevated ·OH yield by ∼2-fold ((Fig. 3G, Fig. S7B)). This enhancement was primarily attributed to Ir^4+^ reduction increasing catalytically active Ir^3+^ sites, which promoted the Fenton reaction. Upon irradiation with an 808 nm laser, the ·OH production was enhanced in a power-dependent manner (Fig. 3H, Fig. S5C). This can be attributed to thermally promoted reaction kinetics, consistent with the Arrhenius equation. Elevated temperatures accelerate both reaction rates and diffusion coefficients, while the associated thermal gradients induce convective flows that further enhance reactant collision frequency [26,37]. To further substantiate this mechanism, we measured ·OH generation at different temperatures and applied the Arrhenius equation to evaluate the temperature dependence of the POD-like reaction catalyzed by IrO_x_-P. The results revealed that higher temperatures markedly accelerated the reaction rate, with an activation energy (Ea) of approximately 4.46 kJ mol^−1^ (Fig. S8), which is comparable to the values reported for highly active metal nanozymes in recent studies [[38], [39], [40]]. Collectively, these results demonstrated that IrO_x_-P catalyzed ·OH production specifically in acidic microenvironments, while photothermal stimulation further enhances ·OH generation in a spatiotemporally controlled manner. Concurrently, efficient GSH depletion sustained radical levels by reducing antioxidant consumption [41,42]. This ternary synergy may maximize tumor ablation efficacy while minimizing collateral damage to healthy tissues.

Furthermore, XPS analysis at multiple time points was performed to monitor the valence state evolution of IrO_x_ upon sequential reactions with GSH and H_2_O_2_. The results revealed that prolonged incubation with GSH led to an increased proportion of Ir^3+^, whereas subsequent addition of H_2_O_2_ decreased the Ir^3+^ fraction while increasing the Ir^4+^ fraction (Figure S9, A-C), confirming a reversible redox cycling between Ir^3+^ and Ir^4+^. UV–Vis absorption spectra of IrO_x_ before and after reaction with GSH or H_2_O_2_ showed no discernible changes (Fig. S9D), and the photothermal heating profiles remained essentially unaltered upon subsequent laser irradiation (Fig. S9E). In addition, ICP-AES measurements demonstrated negligible changes in Ir content before and after reaction: 643.6 μg/mL for IrO_x_, 634.5 μg/mL after reaction with GSH for 30 min, and 653.4 μg/mL after reaction with H_2_O_2_ for 30 min (Table S3). These results indicate that the redox cycling of IrO_x_ does not cause significant particle consumption and does not compromise its subsequent photothermal performance.

### 2.4 In vitro cellular death mediated by IrO_x_-P

Based on the above findings, we evaluated the tumoricidal efficacy of IrO_x_-P. First, cell internalization was investigated by co-incubating 4T1 cells with Cyanine 5 (Cy5)-labeled IrO_x_-P nanoparticles. The red fluorescence within the cells increased with prolonged incubation time (Fig. 3I), indicating that IrO_x_-P nanoparticles were effectively taken up by the cells. CCK-8 assays demonstrated that IrO_x_-P alone induced ∼49.3 % cell death at 200 μg/mL, consistent with its intrinsic ·OH-generating capability via POD/Fenton activities. When combined with 808 nm laser irradiation (0.45 W/cm^2^), the cytotoxic effect of IrO_x_-P was significantly enhanced, with 200 μg/mL of IrO_x_-P resulting in approximately 81.5 % cell death (Fig. 3J). Dose–response curves were fitted to determine the half-maximal inhibitory concentrations (IC_50_) for each treatment group (Fig. S10). The IC_50_ of IrO_x_-P was 211.2 μg/mL, whereas the IC_50_ of the IrO_x_-P + Light group was markedly reduced to 39.41 μg/mL, indicating a substantial enhancement in therapeutic efficacy under photothermal activation. The degree of treatment synergy was further evaluated using the Bliss independence model, yielding a synergy score (S) of 0.393, indicative of a strong synergistic interaction between the photothermal and catalytic effects. Consistently, the modified Combination Index (CI) was calculated as 0.38, which is below the threshold of 1.0, confirming a synergistic therapeutic effect. The enhanced cytotoxicity was further validated by Annexin V/PI flow cytometric analysis (Fig. 3K and L) and Calcein-AM/PI fluorescence staining (Fig. 3M), both of which confirmed increased apoptotic and necrotic cell populations in the combined treatment group. We also tested the cellular oxidative stress induced by IrO_x_-P by using DCFH-DA and HPF probes to detect intracellular ROS and ·OH, respectively. The results showed that IrO_x_-P incubation increased the green fluorescence signals of both DCFH-DA and HPF in cells, and the fluorescence intensity further increased after 808 nm laser irradiation at a low power density (0.45 W/cm^2^) (Fig. 3N–P). This verified that photothermal stimulation potentiated IrO_x_-P's redox catalytic activity within tumor cells.

### 2.5 Photothermal-enhanced ferroptosis-like cellular death

**Fig. 4:**
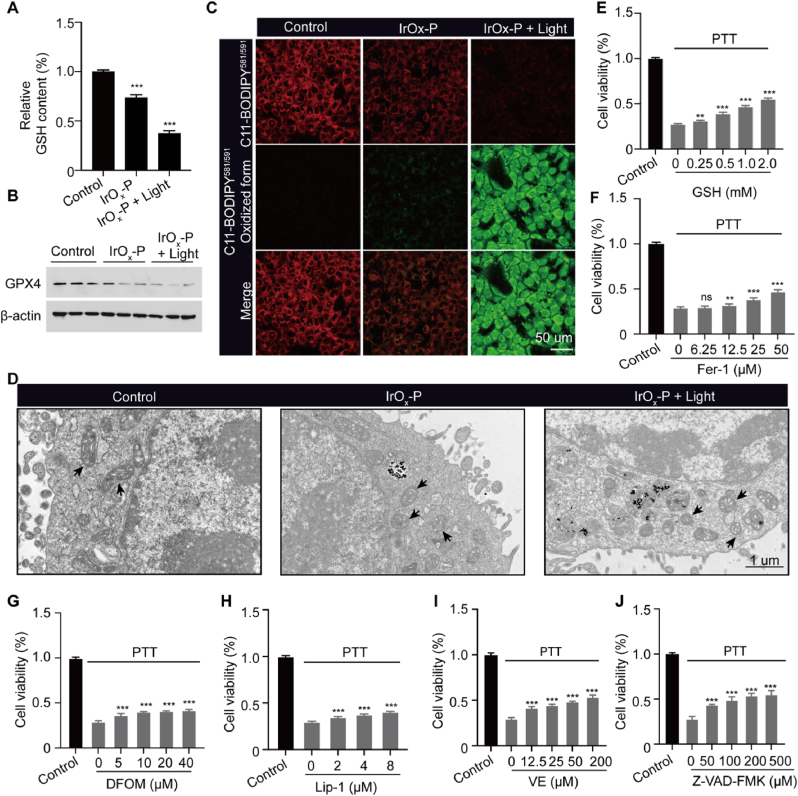
**Intracellular PTT-enhanced ferroptosis. (A)** Relative GSH content in 4T cells after incubation with IrO_x_-P nanoparticles with or without light irradiation. **(B)** Western blot analysis of GPX4 expression levels in 4T1 cells after incubation with IrO_x_-P nanoparticles with or without light irradiation (n = 3). **(C)** Fluorescence images of 4T1 cells stained with C11 BODIPY^581/591^. **(D)** TEM analysis of ferroptosis induction in 4T1 cells with different treatments. Relative cell viability of 4T cells with IrO_x_-P nanoparticles mediated PTT in combination with different concentrations of GSH **(E)**, Fer-1 **(F)**, DFOM **(G)**, Lip-1 **(H),** VE **(I)** and Z-VAD-FMK **(J)**. Data are presented as mean ± s.d. ∗∗P < 0.01, ∗∗∗P < 0.001.

To further delineate the synergistic mechanism of photothermal-induced cell death, we investigated whether IrO_x_-P could effectively deplete intracellular GSH and induce LPO. Treatment with IrO_x_-P alone significantly reduced intracellular GSH levels (by ∼73.5 %), which further declined to ∼37.3 % after low-power-density laser irradiation (Fig. 4A). Western blot analysis confirmed concomitant downregulation of GPX4 expression (Fig. 4B, Fig. S11). LPO was evaluated in vitro using the ratiometric probe C11-BODIPY^581,591^, which, upon oxidation by LPO, exhibited a shift in its maximum excitation/emission peaks from 581/591 nm to 488/510 nm. As shown in Fig. 4C, IrO_x_-P treatment led to the red fluorescence decay and green fluorescence intensification, illustrating a successful LPO induction. Moreover, laser irradiation promoting greater LPO accumulation.

We next evaluated the ability of IrO_x_-P to induce ferroptosis. The morphological changes in mitochondria were first observed using TEM. Compared to the control group, IrO_x_-P incubation and low-power-density laser irradiation led to mitochondrial shrinkage and a reduction in cristae (Fig. 4D). Next, typical ferroptosis-related inhibitors were introduced [34]. GSH, ferrostatin-1 (Fer-1, an inhibitor of ferroptosis), deferoxamine mesylate (DFOM, a specific iron chelating agent), liproxstatin-1 (Lip-1, a LPO scavenging agent) and vitamin E (VE, an antioxidant) could partially rescue the cell death induced by IrO_x_-P-mediated PTT (Fig. 4E–I), whereas inhibitors of autophagy (3-MA) and necrosis (necrostatin-1) showed no such rescuing effect (Figure S12A and B). Furthermore, since both PTT and ROS can induce apoptosis, the apoptosis inhibitor Z-VAD-FMK also partially rescued cell death (Fig. 4J). Notably, quantitative analysis revealed that ferroptosis inhibition restored cell viability by approximately 10–20 %, while apoptosis inhibition restored approximately 30 % of cell death, consistent with the dominant role of PTT/CDT-induced apoptosis but also highlighting a significant ferroptotic contribution to the overall therapeutic outcome. These results above proved that IrO_x_-P as a dual-functional agent capable of synergistically inducing both PTT and ferroptosis. The protective effects of both GSH and VE further demonstrated that GSH depletion and intracellular oxidative stress played pivotal roles in IrO_x_-P-mediated PTT-ferroptosis combination therapy.

### 2.6 Photothermal-driven self-enrichment of IrO_x_-P

**Fig. 5:**
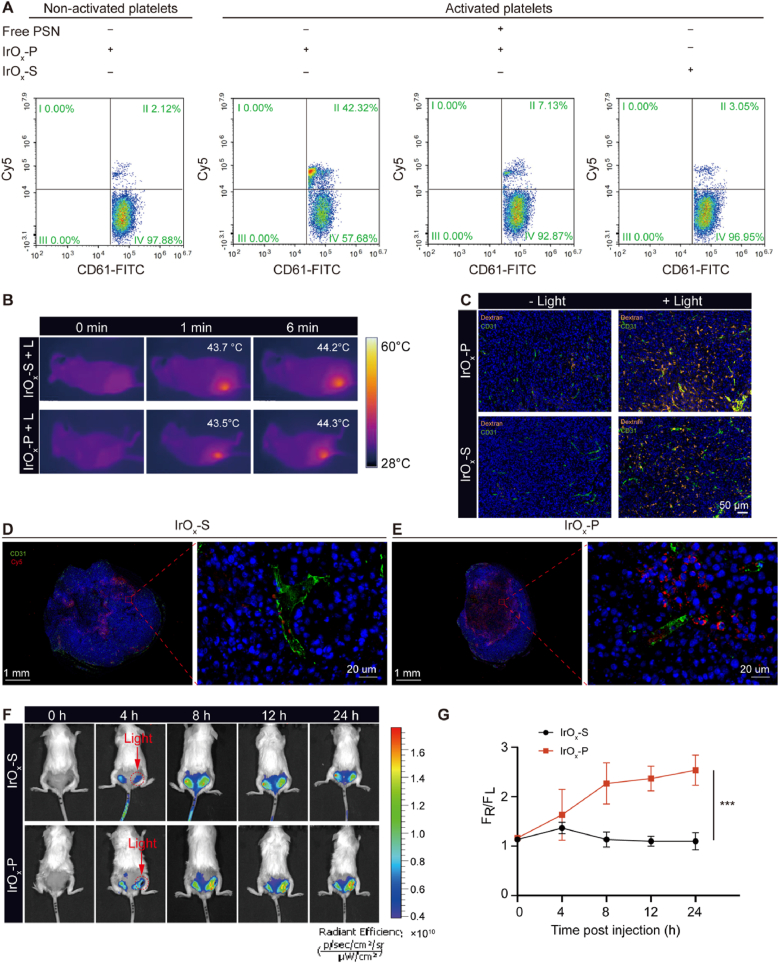
**In vitro and in vivo photothermal-enhanced self-enrichment. (A)** Flow cytometry analysis of IrO_x_-P-Cy5 binding to CD61^+^ platelets under different experimental conditions. Mouse whole blood was collected, and platelets were isolated and either activated with adenosine diphosphate (ADP, 20 μM) or left non-activated. Activated and non-activated platelets were incubated with IrO_x_-P-Cy5 or control IrO_x_-S-Cy5 (PSN sequence scrambled) at 37 °C for 30 min. For competitive inhibition assays, activated platelets were pre-incubated with excess free PSN peptide (100 μg/mL) for 15 min prior to nanoparticle addition. After washing, platelets were stained with anti-CD61-FITC and analyzed by flow cytometry. **(B)** 808 nm laser irradiation-induced temperature rising of local tumor (43–44 °C) for IrO_x_-P-mediated self-enrichment. **(C)** Representative fluorescence images of tumor vessel injury after IrO_x_-S or IrO_x_-P injection with or without laser irradiation assessed by Texas Red-Dextran. Representative immunofluorescence images of tumor sections collected 4 h after mild photothermal irradiation–induced vascular injury, showing CD31 (green), Cy5-labeled IrO_x_-P **(D)** or IrOx-S **(E)** nanoparticles (red), and nuclei (blue, DAPI)**.** In vivo fluorescence imaging **(F)** and quantification **(G)** of bilateral tumor-bearing mice with right tumor irradiation after IrO_x_-P or IrO_x_-S injection. Data are presented as mean ± s.d. ∗∗∗P < 0.001.

Both in vitro and in vivo experiments were conducted to investigate whether IrO_x_-P-mediated PTT could enhance self-enrichment through acute vascular injury. Control nanoparticles (IrO_x_-S) were synthesized by conjugating scrambled peptides (same amino acid composition as PSN but in a scrambled sequence) to verify targeting specificity. We first conducted in vitro experiments to evaluate the targeting ability toward activated platelets. Mouse whole blood samples were activated with adenosine diphosphate (ADP) [43] and subsequently co-incubated with either IrO_x_-P-Cy5 or IrO_x_-S-Cy5 at 37 °C for 30 min. The double-positive rate of IrO_x_-P-Cy5 and CD61-FITC (a platelet-specific antibody) was analyzed using flow cytometry (Figure S13, A-D). Flow cytometry analysis revealed stark differences in platelet targeting efficiency under various conditions (Fig. 5A, S13E). While incubation with non-activated platelet-rich blood showed minimal IrO_x_-P-Cy5^+^/CD61-FITC^+^ double positive cells (1.37 ± 0.70 %), activation with ADP dramatically increased binding rate to 40.69 ± 5.75 %. The specificity of this interaction was confirmed through two key controls: (1) pre-incubation with free PSN competitively inhibited binding, reducing double-positive cells to 7.00 ± 0.56 %, and (2) scrambled-sequence nanoparticles (IrO_x_-S-Cy5) exhibited only baseline binding (2.57 ± 0.49 %) with activated platelets. These results conclusively demonstrated that the platelet targeting ability is both activation-dependent and specifically mediated through PSN recognition. The minimal off-target binding of scrambled controls suggests excellent specificity, a critical safety consideration for clinical translation.

In vivo targeting was evaluated using a bilateral tumor model, in which 4T1 cells were subcutaneously inoculated into the left and right flanks of the mice. The fluorescence intensity of IrO_x_-P-Cy5 in the blood was monitored at different time points after intravenous injection to illustrate its pharmacokinetic profile and determine the optimal irradiation time. The results indicated that the blood concentration of IrO_x_-P-Cy5 dropped sharply after 8 h (Fig. S14). Hence, we performed low-power-density irradiation on the right tumor at 4 h post-injection, maintaining the temperature of right tumors at 43–44 °C for 5 min (Fig 5B, Fig S15). Texas Red-labeled dextran was injected to assess whether vascular damage occurred within the tumor after photoirradiation [22]. Immunofluorescence staining results revealed that for the non-irradiated left tumors, there was almost no dextran extravasation. In contrast, both the IrO_x_-P and IrO_x_-S groups in the right tumors exhibited significant dextran leakage, confirming that the observed vascular disruption resulted from photothermal effects rather than nanoparticle-specific actions (Fig. 5C). CD31 staining was also performed on these same tumor sections; however, as the samples were collected immediately after 30 min of irradiation, no significant change in CD31 signal was observed at this early time point, which is consistent with the notion that structural vascular alterations require a longer period to develop. Since the accumulation of IrO_x_-P or IrO_x_-S in the right tumors at this stage primarily resulted from the EPR effect, there was no significant difference in dextran fluorescence intensity between the two groups.

To further verify the self-reinforcing vascular enrichment of IrO_x_-P, we conducted immunofluorescence staining of CD31 to visualize tumor vasculature 4 h after mild photothermal irradiation–induced endothelial injury. Cy5 fluorescence signals from injected IrO_x_-P-Cy5 or IrO_x_-S-Cy5 nanoparticles were compared. As shown in Fig. 5D and E, both groups exhibited Cy5 accumulation in tumors following light irradiation; however, the IrO_x_-P-Cy5 group displayed substantially higher signal intensity. Importantly, in the IrO_x_-P-Cy5 group, Cy5 fluorescence was observed not only within CD31-positive vascular structures but also in the surrounding perivascular tumor parenchyma. These findings confirm that IrO_x_-P selectively targets photothermal-damaged vascular endothelium and subsequently undergoes secondary accumulation within tumor tissues, thereby achieving self-reinforcing enrichment. Longitudinal in vivo imaging revealed distinct tumor accumulation patterns between IrO_x_-P-Cy5 and IrO_x_-S-Cy5 following photoirradiation. The irradiated right tumors in the IrO_x_-P-Cy5 group exhibited progressively enhanced fluorescence intensity, measuring 2.0-, 2.2-, and 2.3-fold higher than non-irradiated left tumors at 8, 12, and 24 h post-injection (Fig. 5F and G), respectively. This photothermal-enhanced accumulation was completely absent in the IrO_x_-S-Cy5 control group, demonstrating that the observed tumor targeting enhancement specifically required P-selectin-mediated activated platelet binding. To further evaluate the in vivo fate of IrO_x_-P, we performed ex vivo fluorescence imaging of major organs and tumors at 24 h post-injection (Fig. S16). The results revealed that IrO_x_-P fluorescence was predominantly distributed in the kidneys, followed by the liver, indicating that renal clearance is the major metabolic pathway, with partial contribution from hepatobiliary clearance. These results provided direct evidence that mild hyperthermia (43–44 °C) can effectively harness endogenous platelet responses to promote nanoparticle accumulation through controlled vascular injury.

### 2.7 In vivo evaluation of the safety and therapeutic efficacy of IrO_x_-P

**Fig. 6:**
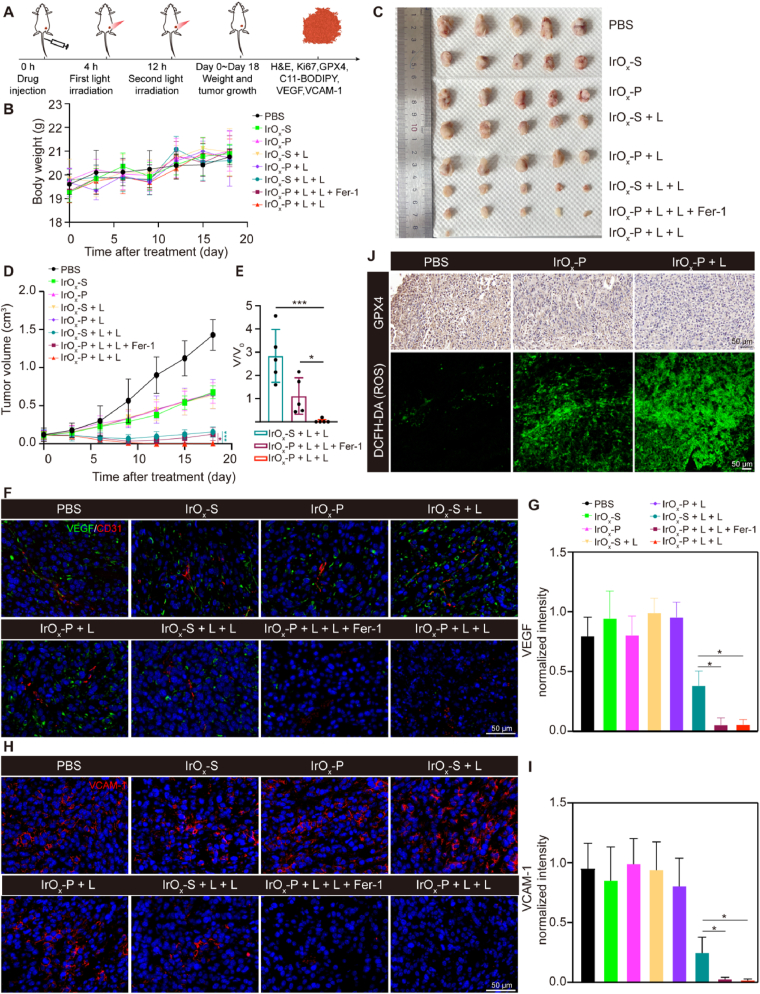
**In vivo therapeutic efficacy evaluation. (A)** Schematic illustration of treatment and efficacy evaluation. **(B)** Mice body weight changes after different treatments (n = 5). Image **(C)** and quantification **(D and E)** of tumor growth after different treatments. Representative immunofluorescent staining images for VEGF, CD31 **(F)** and VCAM-1 **(H)** in tumor tissues after different treatments. Semi-quantitative analysis for expression levels of VEGF **(G)** and VCAM-1 **(I)** (n = 3). **(J)** Immunohistochemical staining of GPX4 and fluorescence staining of DCFH-DA after different treatments. Data are presented as mean ± s.d. ∗∗∗P < 0.001.

Considering the promising targeting capability and therapeutic potential observed in vitro, we next evaluated the in vivo safety and efficacy of IrO_x_-P in a 4T1 tumor mouse model. Following the enrichment kinetics established in Fig. 5F, we implemented an optimized treatment protocol (Fig. 6A). After tumor establishment (10 days post-inoculation), mice received intravenous injections of IrO_x_-P or control IrO_x_-S nanoparticles, followed by two sequential NIR irradiations (808 nm, 0.45 W/cm^2^) - the first at 4 h post-injection (maintaining 43–44 °C for 5 min) and the second 8 h later. Eight experimental groups were carefully designed to dissect the therapeutic mechanisms: (I) PBS control (PBS); (II) IrO_x_-S alone (IrO_x_-S); (III) IrO_x_-P alone (IrO_x_-P); (IV) IrO_x_-S with the first round of irradiation (IrO_x_-S + L); (V) IrO_x_-P with the first round of irradiation (IrO_x_-P + L); (VI) IrO_x_-S with two rounds of irradiation (IrO_x_-S + L + L); (VII) IrO_x_-P with two rounds of irradiation (IrO_x_-P + L + L) and (VIII) IrO_x_-P + L + L plus Fer-1 (IrO_x_-P + L + L + Fer-1) to specifically interrogate ferroptosis involvement.

The in vivo photothermal performance evaluation revealed that IrO_x_-P exhibited significantly enhanced tumor accumulation following the first irradiation, as evidenced by markedly higher intratumoral temperatures compared to IrO_x_-S controls during the second irradiation cycle (Figure S17A and B). Notably, the temperature of IrO_x_-P groups increased to 48 °C within just 5 min of low-power-density irradiation (0.45 mW/cm^2^), demonstrating the combined advantages of nanoparticle self-enrichment and efficient photothermal conversion. The achieved hyperthermia was sufficient to induce tumor cell apoptosis while remaining below the threshold for normal tissue damage, highlighting the therapeutic safety window enabled by this targeted approach.

During the treatment period, the body weight and tumor volume of the mice were monitored over 18 days. The body weight of the mice increased gradually, with no significant differences observed among the groups, indicating that all treatments had negligible adverse effects on the health of the mice (Fig. 6B). While PBS controls showed rapid tumor progression, moderate growth inhibition was observed in IrO_x_-S, IrO_x_-P, IrO_x_-S + L, and IrO_x_-P + L groups, attributable to intrinsic ROS-mediated apoptosis/ferroptosis (Fig. 6C–E). Dual irradiation groups (IrO_x_-S + L + L and IrO_x_-P + L + L) exhibited significantly enhanced tumor suppression, with IrO_x_-P + L + L demonstrating superior efficacy due to PSN-mediated self-enrichment. This therapeutic advantage was partially reversed by Fer-1 co-treatment, validating ferroptosis involvement. Tumor tissues were harvested after treatment for pathological analyses. Consistent with the tumor growth curves, H&E staining revealed varying degrees of tumor damage across the treatment groups, with the IrO_x_-P + L + L group exhibiting the maximum tumor necrosis. Ki67 staining also showed lowest proliferation rates (Fig. S18). Further mechanistic investigation revealed that IrO_x_-P + L + L treatment potently disrupted tumor vasculature and suppressed neovascularization, as evidenced by significant downregulation of CD31, vascular endothelial growth factor (VEGF), and vascular cell adhesion molecule-1 (VCAM-1) expression as compared with IrO_x_-S + L + L treatment (Fig. 6F–I). Moreover, photoirradiation synergistically enhanced the downregulation of GPX4 expression and the generation of ROS in tumor tissues induced by IrO_x_-P (Fig. 6J, Fig. S19). These results collectively established IrO_x_-P as a potent treatment platform that coupled light-triggered self-enrichment with synergistic CDT-ferroptosis.

Comprehensive biosafety evaluation confirmed the excellent biocompatibility of IrO_x_-P. Hemolysis assays demonstrated negligible red blood cell damage, while blood biochemistry and complete blood count analyses revealed no significant alterations in key parameters (Figure S20, A-C), even following dual irradiation treatment. Histopathological examination of major organs (heart, liver, spleen, lungs, and kidneys) showed normal tissue architecture without signs of inflammation, necrosis, or other pathological changes (Fig. S20D). In addition, we specifically examined the local skin at the tumor irradiation site to assess potential thermal injury caused by the self-enriching photothermal therapy. Photographs and H&E staining revealed that 7 days post-irradiation, the skin displayed a localized scab, with histology showing thickened collagen fiber bundles (Fig. S21A and C). By day 18 post-treatment, the skin morphology had returned to normal, comparable to untreated skin (Fig. S21B and D), indicating that the transient thermal changes were reversible. These findings collectively establish IrO_x_-P as a safe nanotherapeutic platform with outstanding tissue and blood compatibility for potential clinical translation.

### 2.8 H_2_O_2_-activated PA imaging of IrO_x_-P

**Fig. 7:**
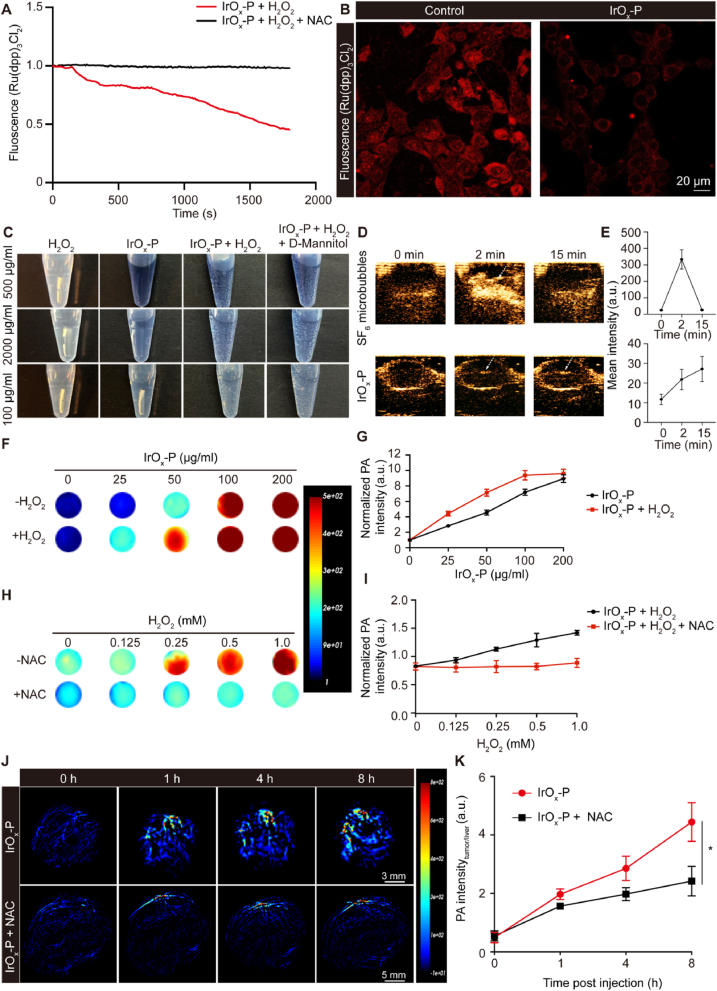
**In vitro and in vivo H_2_O_2_-activated PA imaging. (A)** O_2_ generation kinetics of IrO_x_-P assessed by the fluorescence-quenching probe [Ru(dpp)_3_] Cl_2_ (Ex/Em: 450/610 nm). **(B)** Representative fluorescence images of O_2_ generation in 4T1 cells with IrO_x_-P incubation. **(C)** Photographs of O_2_ generation after adding H_2_O_2_ with different concentrations of IrO_x_-P. D-Mannitol is a ·OH scavenger. **(D)** In vivo ultrasound imaging of tumors after intratumoral injection of SF_6_ microbubbles or IrO_x_-P (100 μg/mL) nanoparticles. **(E)** Quantification of ultrasound signal intensity changes in tumors following intratumoral injection of SF_6_ microbubbles or IrO_x_-P (n = 3). Representative PA images **(F)** and signal intensity **(G)** of different concentrations of IrO_x_-P in the presence/absence of H_2_O_2_ (1 mM) (n = 3). Representative PA images **(H)** and signal intensity **(I)** of IrO_x_-P (100 μg/mL) with different concentrations of H_2_O_2_ in the presence/absence of NAC (10 mM) (n = 3). In vivo PA imaging **(J)** and corresponding intensity of liver **(K)** in 4T1 tumor-bearing mice after intravenous injection of IrO_x_-P (10 mg/kg) with or without NAC pretreatment (n = 3). Data are presented as mean ± s.d. ∗P < 0.05.

PA imaging, which combines the deep tissue penetration of ultrasound with the high sensitivity of optical imaging, has emerged as a powerful tool for tumor diagnosis in recent years. Notably, O_2_ microbubbles have been reported to enhance PA signals via microbubble-mediated non-inertial cavitation, thereby improving imaging contrast [27]. Given that IrO_x_ possesses intrinsic photothermal properties, it can inherently serve as a PA contrast agent. In addition, its CAT-like activity enables the catalytic decomposition of tumor-overexpressed H_2_O_2_ into O_2_. We therefore hypothesized that IrO_x_-P could react with intratumoral H_2_O_2_ to generate O_2_ microbubbles, thereby amplifying PA signals through a combination of intrinsic photothermal conversion and TME–responsive catalytic O_2_ production. We first confirmed that the addition of H_2_O_2_ did not alter the absorption spectrum of IrO_x_-P, indicating good optical stability under TME-mimicking conditions (Fig. S9D). We next validated IrO_x_-P's CAT-like activity using an O_2_ sensitive-fluorescent probe (Ru(ddp)_3_Cl_2_ [27]. The rapid fluorescence quenching upon H_2_O_2_ exposure and its reversal by N-acetylcysteine (NAC) scavenging confirmed H_2_O_2_-triggered O_2_ generation (Fig. 7A). This H_2_O_2_-responsiveness was maintained in 4T1 cells (Fig. 7B), demonstrating TME-responsive activation. Direct observation showed prominent microbubble formation when IrO_x_-P was reacted with H_2_O_2_, whereas neither H_2_O_2_ nor IrO_x_-P alone produced visible bubbles. Moreover, the addition of the ·OH scavenger D-mannitol did not affect bubble generation, indicating that the bubbles were predominantly composed of O_2_ rather than products arising from ·OH-mediated reactions (Fig. 7C). We next employed ultrasound imaging to monitor intratumoral O_2_ generation in vivo. Following intratumoral injection of IrO_x_-P, a diffuse enhancement in ultrasound signals was observed, distinct from the strong, localized signals produced by direct injection of SF6 microbubbles, suggesting a slow and sustained O_2_ generation process in the tumor (Fig. 7D and E).

Given the sustained O_2_ production demonstrated above, we subsequently investigated its contribution to PA signal enhancement. The PA signals of IrO_x_-P elevated with its concentration and significantly enhanced in the presence of H_2_O_2_ (Fig. 7F and G). Moreover, The PA signals also increased in a H_2_O_2_ concentration-dependent manner. Upon pre-treatment of NAC to scavenge H_2_O_2_, the PA signals significantly decreased (Fig. 7H and I). In vivo studies in 4T1 tumor-bearing mice showed time-dependent PA signal intensification, which was significantly reduced by NAC pretreatment (Fig. 7J and K) while liver signals remained stable (Fig. S22). These findings demonstrate a dual PA imaging mechanism of IrO_x_-P that integrates intrinsic photothermal conversion with H_2_O_2_-activated catalytic O_2_ generation. Compared with many existing PA contrast enhancement strategies that encapsulate imaging agents within preformed microbubbles, which often require complex fabrication and suffer from short imaging windows due to gas dissolution or bubble collapse [44,45], our approach leverages the intrinsic, sustained O_2_ generation of IrO_x_-P in response to the tumor microenvironment. This design not only enables TME-responsive signal amplification but also prolongs the effective imaging window, thereby offering sustained imaging capability.

## 3 Conclusion

In summary, we have developed a photothermal-cascaded IrO_x_-P nanozyme with dual responsiveness to external irradiation and the tumor microenvironment, enabling efficient and coordinated CDT/ferroptosis therapy and enhanced PA imaging. This system circumvents the complexity and inefficiency of conventional multi-component assemblies by enabling photothermally triggered platelet-mediated targeting and a self-reinforcing catalytic cascade through Ir^3+^/Ir^4+^ redox cycling. The dual-responsive activation achieves efficient GSH depletion and ·OH generation, ultimately leading to GPX4 inactivation and lethal LPO. Beyond its local therapeutic efficacy, the intrinsic features of ROS burst, GPX4 suppression, and lethal LPO, together with localized hyperthermia induced by photothermal therapy, suggest a strong potential to induce immunogenic cell death (ICD) through both ferroptotic and thermal damage pathways [[46], [47], [48], [49]]. These mechanisms are considered conductive to dendritic cell activation and antigen presentation, thereby reshaping tumor immune microenvironment and augmenting immune cell infiltration [[50], [51], [52], [53], [54]]. These immunological implications warrant future investigation into the immunomodulatory capacity of IrOx-P, particularly in combination with immune checkpoint inhibitors or cancer vaccines, to further advance its clinical translation.

## 4 Experimental section

### 4.1 Materials

Ircl_3_, NH_2_-PEG_2000_-COOH, sodium citrate, DTNB, GSH, Fer-1 and NAC were purchased from Aladdin Reagent Co., Ltd. (Shanghai, China). PSN was synthesized by GOTOP Biotechnology Co., Ltd. (Hangzhou, China). Sulfo-Cy5-NHS was purchased from DUOFLUOR Technology, Inc. (Wuhan, China). TMB, MB and Lip-1 were purchased from Macklin Biochemical Co. Ltd. (Shanghai, China). Vitamin E, Necrostatin-1, 3-MA, DFOM, Z-VAD-FMK and ADP were obtained from Yuanye Bio-Technology Co., Ltd. (Shanghai, China). DCFH-DA, HPF and C11-BODIPY^581/591^ were obtained from Maokang Biotechnology Co., Ltd. (Shanghai, China). CCK-8 and GSH Assay Kit were purchased from Beyotime Institute of Biotechnology (Jiangsu, China). Calcein AM/PI Assay Kit was purchased from Yeasen Biotechnology Co., Ltd. (Shanghai, China). Texas Red-Dextran-70,000 KD was obtained from Thermo Fisher Scientific Inc. (Waltham, USA). Anti-GPX4 antibody (T56959S) was obtained from Abmart Inc. (Shanghai, China). FITC anti-CD61 antibody (104305) was obtained from Biolegend Inc. (Shanghai, China). Anti-CD31 antibody, Anti-VEGF antibody and Anti-VCAM-1 antibody were purchased from Servicebio Co., Ltd. (Wuhan, China).

### 4.2 Synthesis of IrO_x_-P

IrO_x_ nanoparticles were synthesized by dissolving IrCl_3_ in water and stirring the solution at room temperature overnight. The solution was then refrigerated for 3 days, followed by filtration through a membrane filter (0.22 μm). Sodium citrate (mass ratio 1:3) was added to the filtered IrCl_3_ solution, and the pH was adjusted to 10 using NaOH. The mixture was vigorously stirred at 100 °C, with pH monitored and readjusted to 10 every 30 min until stabilization. After pH stabilization, stirring was continued for an additional 2 h to obtain IrO_x_ nanoparticles.

To conjugate the peptide, the IrO_x_ nanoparticles were modified with PEG_2000_. 70 mg of IrO_x_ powder was ultrasonically dispersed in 30 mL of dimethylformamide (DMF), followed by the dropwise addition of 280 μL of (3-aminopropyl) triethoxysilane (APTES). The mixture was stirred at 80 °C in an oil bath for 8 h. The product was washed three times with DMF (13,000 rpm, 10 min each) and three times with ethanol, then freeze-dried for further use. Next, 0.5 g of NH_2_-PEG_2000_-COOH (0.25 mmol) was mixed with 0.1 g of EDC (0.5 mmol) and 0.15 g of NHS (1.25 mmol) in 5 mL of DMF, followed by ultrasonic dispersion for 30 min. The mixture was stirred in the dark for 12 h, after which 10 mg of IrO_x_-NH_2_ was added and the reaction continued for 3 days. The product was dialyzed for 48 h and freeze-dried to obtain IrO_x_-PEG.

For peptide conjugation, 10 mg of PSN, 150 mg of NHS, and 100 mg of EDC were dissolved in 5 mL of DMF and stirred in the dark for 4 h. The activated peptide solution was then added dropwise to 1 mL of IrO_x_-PEG aqueous solution (10 mg/mL), followed by continuous stirring in the dark for 12 h. The final product, IrO_x_-P, was obtained by dialyzing the mixture using a 10 kDa molecular weight cutoff dialysis membrane for 48 h to remove unreacted reagents and byproducts.

### 4.3 Characterization of IrO_x_-P

The morphology and size of the nanoparticles were observed and measured using TEM (JEM-F200), while the elemental composition was analyzed through EDS mapping. The optical absorption was measured using a full-spectrum microplate reader (SyneryH1). The chemical composition was evaluated by XPS (K-Alpha), and the successful conjugation of PSN was confirmed by FTIR (Nicolet iS20). The hydrodynamic size and zeta potential were determined using a Malvern Zetasizer (Nano ZS90). Photothermal temperatures were monitored by a thermal camera (Fotric 223s). Ir content was detected via ICP-AES.

### 4.4 In vitro photothermal effect of IrO_x_-P

The aqueous solution of IrO_x_-P was irradiated with a 808 nm laser of different power densities for 15 min, and the temperature rise curves were recorded using a thermal camera. For photothermal stability testing, the solution was irradiated for 300 s (1.5 W/cm^2^), followed by another 300 s of natural cooling. This heating-cooling cycle was repeated five times. For photothermal conversion efficiency testing, the solution was irradiated for 300 s (1.5 W/cm^2^), after which the laser was turned off to allow cooling to the initial temperature. The temperature change curve was recorded and the photothermal conversion efficiency was calculated as below [55]:

$$\eta =\frac{hS(T_{\max}-T_{surr)-Q_{sys}}}{I\left(1-10^{{-A}_{\lambda }}\right)}$$

where hS is the heat transfer coefficient, Q_sys_ is the heat dissipated from light absorbed by the solvent and sample cell, I is the incident laser power, and A_λ_ is the absorbance of the sample at 808 nm. Baseline corrections were performed using water under identical experimental conditions, and the absorbance values were measured using a UV–vis spectrophotometer.

### 4.5 GSH depletion assay

The concentration of GSH was determined by measuring the absorbance of DTNB at 412 nm. Briefly, 1 mM GSH was mixed with 100 μg/mL IrO_x_-P solution and incubated at different pH values (7.4, 5.5, and 4.0) for 60 min. After centrifugation at 13,000 rpm for 10 min, 100 μL of the supernatant was collected and mixed with 0.5 mM DTNB to measure the remaining GSH. To assess the impact of photothermal effects on GSH depletion, 1 mM GSH was mixed with 100 μg/mL IrO_x_-P solution (pH 4.0) and irradiated with an 808 nm laser at different power densities for 10 min. The supernatant was detected as previously described.

### 4.6 POD-like activity

Under H_2_O_2_ conditions, TMB was selected as the chromogenic substrate to evaluate the POD-like activity. 6 μL of TMB ethanol solution (10 mg/mL) was added to 984 μL of IrO_x_-P aqueous solution (100 μg/mL) at different pH values (4.0, 5.5, 6.5 and 7.4) containing 10 μL of H_2_O_2_ (30 mM). After reacting at room temperature for 10 min, the absorbance was measured at 652 nm. To evaluate the relationship between POD-like activity and the concentrations of H_2_O_2_ and IrO_x_-P, 6 μL of TMB (10 mg/mL) was added to 984 μL of IrO_x_-P, solutions at varying concentrations, followed by the addition of 10 μL of H_2_O_2_ at different concentrations.

For quantitative kinetic analysis, IrO_x_-P dispersions at a fixed concentration (50 μg/mL) were incubated in 0.2 M acetate buffer (pH 4.0) with varying concentrations of H_2_O_2_ and 0.8 mM TMB at 37 °C. The reaction was initiated by adding H_2_O_2_ and allowed to proceed for 3 min. The absorbance of oxidized TMB (oxTMB) was recorded at 652 nm, and initial reaction rates (V_0_) were calculated from the linear portion of the absorbance–time curve using the molar extinction coefficient of oxTMB (*ε* = 39,000 M^−1^ cm^−1^). Michaelis–Menten kinetic parameters (K_m_) were determined by fitting the data to the Michaelis–Menten equation and confirmed by Lineweaver–Burk double reciprocal plots.

### 4.7 OH generation assay

IrO_x_-P solution (100 μg/mL) was added with H_2_O_2_ (10 mM), MB (10 μg/mL) and NaHCO_3_ (25 mM) to react for 20 min at different pH value (7.4 and 5.5). After centrifugation at 13,000 rpm for 10 min, 100 μL of the supernatant was collected and detected at the absorbance at 665 nm. For GSH-assisted ·OH generation detection, IrO_x_-P solution was first interacted with GSH (1 mM) at pH value of 5.5 for 30 min. To assess the impact of photothermal effects on ·OH generation, the solution was irradiated with an 808 nm laser at different power densities for 10 min. Moreover, 30 μl DMPO (100 mM) was added to 30 μl of sample for ESR assay.

The temperature dependence of the·OH generation catalyzed by IrO_x_-P nanoparticles was assessed using MB degradation. IrO_x_-P nanoparticles (100 μg/mL) were incubated with H_2_O_2_ (10 mM) in acetate buffer (pH 4.0) at different temperatures (25, 35, 45, and 55 °C). The decrease in MB absorbance at 665 nm was monitored over time using a UV–vis spectrophotometer, and the initial degradation rate (*k*) was calculated from the linear portion of the absorbance-time curve. The natural logarithm of the rate constant (ln *k*) was plotted against the reciprocal of absolute temperature (1/*T*, in K) to obtain the Arrhenius plot. The apparent activation energy (*E*_*a*_) was calculated from the slope (–*E*_*a*_/R) according to the Arrhenius equation:

$$\ln\;k=\ln\;A-\frac{E_{a}}{RT}$$

where *A* is the pre-exponential factor, *R* is the universal gas constant (8.314 J mol^−1^ K^−1^), and *T* is the absolute temperature.

### 4.8 Cellular culture and uptake

4T1 cells were cultured in RPMI-1640 medium supplemented with 10 % fetal bovine serum (FBS) and 1 % penicillin-streptomycin at 37 °C under a 5 % CO_2_ atmosphere. 4T1 cells were cultured in confocal dishes and were incubated with Cy5 labeled IrO_x_-P nanoparticles at a concentration of 100 μg/mL. After co-culturing for 0 h, 4 h, and 8 h, the cells were fixed with 4 % PFA, and the nuclei were stained with DAPI. Cellular uptake was visualized and captured using a confocal microscope (LSM 880, Zeiss).

### 4.9 In vitro cytotoxicity

4T1 cells were seeded in 96-well plates (10^4^ cells/well) and cultured in complete RPMI-1640 medium for 24 h. Subsequently, the cells were incubated with different concentrations of IrO_x_-P (0–200 μg/mL) for 8 h. After 8 h, the drug-containing medium was replaced with fresh medium. For the IrO_x_-P + Light group, cells were irradiated with an 808 nm laser (0.45 W/cm^2^) for 10 min. After 2 h, cell viability was assessed using the CCK-8 assay and the absorbance at 450 nm was measured by a microplate reader (SyneryH1). Furthermore, the treated cells were also stained with Calcein-AM/PI and imaged by a confocal microscope. IC_50_ values were obtained by fitting the dose–response curves to a four-parameter logistic model. The modified combination index (CI) was calculated as:

$${CI}_{modified}=\frac{{IC50}_{combination}}{{IC50}_{IrOx-P}}$$

since Light alone showed no measurable cytotoxicity and thus IC_50_ could not be determined. Synergy was also evaluated using the Bliss independence model:

$$E_{expected}=E_{IrOx-P}+E_{Light}-E_{IrOx-P}\times E_{Light}$$

$$S=E_{IrOx-P+Light}-E_{expected}$$

S > 0 indicates synergy, S = 0 additivity, and S < 0 antagonism.

### 4.10 Cellular ROS generation assay

4T1 cells were cultured in confocal dishes and incubated with IrO_x_-P (100 μg/mL) for 8 h. For the IrO_x_-P + Light group, cells were irradiated with an 808 nm laser (0.45 W/cm^2^) for 10 min. The culture medium was replaced by fresh DCFH-DA- or HPF-containing medium (10 μM) for 30 min. The fluorescence of DCFH-DA or HPF were visualized by a confocal microscope with Ex/Em of 488 nm/525 nm.

### 4.11 Cellular GSH and GPX4 assay

4T1 cells were cultured in 10 cm dishes and incubated with IrO_x_-P (100 μg/mL) for 8 h. For the IrO_x_-P + Light group, cells were irradiated with an 808 nm laser (0.45 W/cm^2^) for 10 min. The GSH concentration was evaluated using GSH assay kit according to manufacturer's instruction. For GPX4 expression evaluation, the total proteins were extracted from treated cells through RIPA containing 1 % of protease inhibitors and quantified using a BCA assay. Proteins were separated by SDS-PAGE, transferred to a PVDF membrane, and incubated with primary and secondary antibodies for GPX4 detection.

### 4.12 Cellular LPO detection

4T1 cells were cultured in confocal dishes and exposed to different treatments. Then, the culture medium was replaced by fresh C11-BODIPY^581/591^-containing medium (10 μM) for 30 min. Nonoxidized and oxidized LPO were detected by monitoring the fluorescence shift of C11-BODIPY from red (591 nm, non-oxidized) to green (510 nm, oxidized) using a confocal microscope.

### 4.13 TEM analysis for ferroptosis

To evaluate ferroptosis at the ultrastructural level, treated cells were collected and fixed with 2.5 % glutaraldehyde in 0.1 M phosphate buffer (pH 7.4) for 2 h at 4 °C. The samples were then post-fixed with 1 % osmium tetroxide for 1 h, dehydrated through a graded ethanol series (50 %, 70 %, 90 %, and 100 %), and embedded in epoxy resin. Ultrathin sections (70–90 nm) were cut using an ultramicrotome and stained with uranyl acetate and lead citrate. The sections were examined using TEM to observe morphological changes characteristic of ferroptosis, such as mitochondrial shrinkage, reduced cristae, and increased membrane density.

### 4.14 Cellular ferroptosis, apoptosis, necroptosis and autophagy inhibition assay

4T1 cells were seeded in 96-well plates (10^4^ cells/well) and cultured in complete RPMI-1640 medium for 24 h. Next, cells were pre-incubated with various inhibitors (as indicated in Fig. 4 and Fig. S5) for 4 h, followed by the addition of IrO_x_-P and further incubation for 8 h before irradiation. After irradiation, the cells were cultured for an additional 2 h, and cell viability was assessed using the CCK-8 assay.

### 4.15 In vitro binding test of activated platelets

1.5 mL plastic tube were pre-coated with sodium citrate anticoagulant, and 100 μL of blood was collected from the mouse retro-orbital venous plexus. ADP was added to the blood at a final concentration of 20 μM and incubated at 37 °C for 30 min to activate platelets. For non-activated platelets, Cy5-labeled IrO_x_-P was added, while for activated platelets, either Cy5-labeled IrO_x_-P or IrO_x_-S was added. For the PSN peptide competition inhibition test group, the blood was pre-incubated with PSN (100 μg/mL) for 30 min before adding Cy5-labeled IrO_x_-P. After incubation, CD61-FITC antibody was added, and the samples were analyzed using flow cytometry.

### 4.16 Animal model

Female BALB/c mice were obtained from Beijing HFK Bioscience Co., Ltd. All animal studies conformed to the guidelines of the Animal Care Ethics Commission of Kunming Medical University. To establish 4T1 tumor models, 100 μL of 4T1 cell suspension (1 × 10^6^ cells) in 1 × PBS was injected into the right flank (unilateral tumor model) or right and left flanks (bilateral tumor model) of each mouse.

### 4.17 In vivo pharmacokinetic analysis

Cy5-labeled IrO_x_-P nanoparticles (10 mg/kg) were intravenously injected into BALB/c mice. The blood was collected through retro-orbital venous plexus at 2, 4, 8, 12 and 24 h after injection. The blood samples were then centrifuged at 1000 g for 10 min and detected by a microplate reader to determine the concentration of IrO_x_-P.

### 4.18 In vivo acute vascular injury assessment

Bilateral 4T1 tumor-bearing mice were intravenously injected with IrO_x_-P nanoparticles (10 mg/kg). Four hours post-injection, the right tumors were irradiated with an 808 nm laser to maintain the tumor surface temperatures at 43–44 °C for 5 min. At 8 h after injection, the mice were intravenously administered 100 μL of Texas Red-Dextran-70,000 KD (25 mg/mL). 30 min later, the mice were euthanized, and the tumors were harvested for CD31 immunofluorescence staining.

### 4.19 In vivo self-enrichment assay

IrO_x_-P nanoparticles (10 mg/kg) were intravenously administered to mice bearing bilateral 4T1 tumors. Four hours later, the right tumors were subjected to 808 nm laser irradiation, keeping the tumor surface temperature at 43–44 °C for 5 min. The fluorescence changes were monitored by a in vivo imaging system (IVIS LuminaIII) at 0, 4, 8, 12 and 24 h. The ratio of fluorescence intensity between the right and left tumors was analyzed. In addition, major organs were harvested and imaged to investigate the organ distribution of IrO_x_-P nanoparticles.

### 4.20 In vivo photothermal effect

Unilateral tumor-bearing mice were intravenously injected with IrO_x_-P nanoparticles (10 mg/kg). For mice subjected to one-stage irradiation (PBS + L, IrO_x_-S + L and IrO_x_-P + L), the 808 nm laser was used at 4 h post-injection to maintain the tumor surface temperatures at 43–44 °C for 5 min, with no second round of irradiation performed. For two-stage irradiation groups (IrO_x_-S + L + L and IrO_x_-P + L + L), in addition to the first round of irradiation to induce vascular injury, the tumor sites were irradiated with an 808 nm laser for 10 min at 12 h post-injection to kill tumor cells. During the second round of irradiation, the temperature changes in the mouse tumors were recorded using a thermal imaging camera.

### 4.21 In vivo therapeutic effect

Unilateral tumor-bearing mice were randomly divided into eight groups (n = 5) and intravenously injected with PBS, IrO_x_-S (10 mg/kg) or IrO_x_-P (10 mg/kg). For the one-stage groups (IrO_x_-S + L and IrO_x_-P + L), mice received the first round of irradiation only at 4 h post-injection (808 nm, 43–44 °C for 5 min). For the two-stage groups (IrO_x_-S + L + L, IrO_x_-P + L + L and IrO_x_-P + L + L + Fer-1), Mice underwent the second round of irradiation (808 nm, 0.45 W/cm^2^, 10 min) at 12 h post-injection, following the first round of irradiation. To confirm ferroptosis induction, Fer-1 was administered via intraperitoneal injection on 0 and 7 days after light irradiation to mice in IrO_x_-P + L + L + Fer-1 group. The body weight and tumor volume were recorded every 3 days for 18 days.

### 4.22 Histological analysis of tumors

At 18 days post-treatment, tumors were harvested and fixed in 4 % PFA. The direct tumor-killing and proliferation-inhibiting effects of the treatment were assessed using hematoxylin and eosin (H&E) staining and Ki67 immunohistochemistry. Immunofluorescence staining of CD31, VEGF and VCAM-1 were performed to analyze vascular injury.

### 4.23 In vivo ROS generation and GPX4 deactivation

The tumor-bearing BALB/c mice were divided into three groups randomly: PSB treatment group, IrO_x_-P intravenous injection group and IrO_x_-P intravenous injection with one-stage irradiation group. For in vivo ROS assay, fresh tumors were rapidly frozen in liquid nitrogen and sectioned into 10 μm thick slices using a cryostat. The sections were stained with the DCFH-DA probe (10 μM) and imaged under a confocal microscope. For GPX4 expression level evaluation, tumors were harvested and fixed in 4 % PFA. Immunohistochemical staining of GPX4 were performed using standard protocols. For quantitative analysis, images of representative fields were captured under identical optical conditions using a bright-field microscope. GPX4-positive staining intensity, positive area percentage, and overall IHC score were evaluated using ImageJ with the IHC Profiler plugin. The IHC score was calculated as the sum of the staining intensity score (0–3) and the proportion score (0–4), yielding a total score range of 0–7. All evaluations were performed by two independent blinded observers.

### 4.24 In vivo biosafety

To evaluate the hemocompatibility of IrO_x_-P nanoparticles, a hemolysis test was performed. Briefly, fresh mouse blood was collected in anticoagulant-treated tubes and centrifuged at 1500 rpm for 10 min to isolate red blood cells (RBCs). The RBCs were washed three times with PBS and resuspended in PBS to prepare a 2 % (v/v) RBC suspension. IrO_x_-P nanoparticles at various concentrations (0–100 μg/mL) were incubated with the RBC suspension at 37 °C for 3 h. After incubation, the samples were centrifuged at 1500 rpm for 10 min, and the absorbance of the supernatant was measured at 540 nm using a microplate reader. Deionized water was used as positive control. To evaluate the potential toxicity on liver and kidney function in mice undergoing two-round irradiation therapy, blood samples were collected at 18 days for routine blood tests and blood biochemistry analyses. Moreover, major organs and skins were harvested and assessed for potential damage using H&E staining.

### 4.25 O_2_ generation assay

IrO_x_-P nanoparticles (100 μg/mL) were incubated with H_2_O_2_ (1 mM), and Ru(ddp)_3_Cl_2_ probe (1 mg/mL) was added to detect O_2_ generation. The fluorescence intensity change at 613 nm was recorded for 30 min. For comparison, H_2_O_2_ was pretreated with the ROS scavenger NAC (10 mM) for 5 min. Cellular O_2_ generation was assessed by adding Ru(ddp)_3_Cl_2_ probe (10 μM) to IrO_x_-P-treated 4T1 cells. The formation of O_2_ microbubbles during the reaction was observed and recorded using a camera. To further investigate the role of ·OH in the catalytic process, D-mannitol (10 mM), a hydroxyl radical scavenger, was added to the IrO_x_-P/H_2_O_2_ reaction mixture prior to assay initiation.

### 4.26 In vivo ultrasound imaging

To visualize oxygen microbubble formation within tumors, in vivo ultrasound contrast imaging was performed. For contrast control, sulfur hexafluoride (SF_6_) microbubbles (SonoVue®, Bracco, Italy) were reconstituted in 5 mL sterile saline according to the manufacturer's protocol. A volume of 50 μL of the freshly prepared suspension was intratumorally injected under real-time ultrasound guidance. For nanoparticle evaluation, IrO_x_-P was administered intratumorally at a dose of 10 mg kg^−1^ in a total volume of 50 μL. Ultrasound imaging was carried out using a Vetus 9 Pro ultrasound system (Mindray, China) in contrast-enhanced imaging mode, and the dynamic generation and distribution of microbubbles within the tumor tissue were recorded immediately after injection.

### 4.27 In vitro and in vivo PA imaging

To evaluate the O_2_-enhanced PA imaging mediated by IrO_x_-P, PA signals of IrO_x_-P nanoparticles with different concentration (0–200 μg/mL) in the presence or absence of H_2_O_2_ (1 mM) were recorded using MarsSonics PIIP Photoacoustic Integrated Imaging Platform. To test the relationship between H_2_O_2_ concentration and PA signals, IrO_x_-P (100 μg/mL) treated with different concentration of H_2_O_2_ (0–1 mM) were subjected to PA imaging by using MarsSonics PIIP. Moreover, NAC (10 mM) was used to scavenge H_2_O_2_ to determine the role of H_2_O_2_ in enhancing PA signal. For in vivo PA imaging, tumor-bearing mice were intravenously injected with IrO_x_-P (10 mg/kg), and photoacoustic imaging was performed at 0, 1, 4, and 8 h post-injection. For comparison, mice were pre-treated with an intraperitoneal injection of NAC (500 mg/kg) 2 h before iridium oxide administration, followed by photoacoustic imaging at 0, 1, 4, and 8 h post-injection. Liver PA signal was used as a normalization control.

### 4.28 Statistical analysis

All data are presented as the means ± SDs. Statistical comparisons were evaluated by one-way ANOVA or two-way ANOVA. P < 0.05 was considered statistically significant. Significant p values are denoted by ∗p < 0.05, ∗∗p < 0.01 and ∗∗∗p < 0.001.

## CRediT authorship contribution statement

**Xi Zhu:** Writing – review & editing, Writing – original draft, Visualization, Methodology, Investigation, Funding acquisition, Formal analysis, Conceptualization. **Yang Zhang:** Writing – original draft, Methodology, Funding acquisition, Formal analysis. **Yufei He:** Methodology, Formal analysis, Data curation. **Li Li:** Writing – review & editing, Methodology. **Xiaofei Luo:** Formal analysis, Data curation. **Ran Zhao:** Methodology, Data curation. **Xiaoying Yan:** Writing – review & editing, Methodology, Investigation. **Ceshi Chen:** Writing – review & editing, Supervision, Investigation, Funding acquisition, Conceptualization.

## Funding

This work was supported by Noncommunicable Chronic Diseases-10.13039/501100018537National Science and Technology Major Project (2023ZD0502200), Yunnan Fundamental Research Projects (202301AT070272, 202501AS070023, 202301AY070001-168, 202201BC070002), 10.13039/501100001809National Science Foundation of China (82460369, U2102203 and 82430084), Biomedical Projects of Yunnan Key Science and Technology Program (202302AA310046), Yunnan Academician Expert Workstation (202505AF350058), the Innovative Research Team of Yunnan Province (202405AS350016), the Taishan Scholars Program of Shandong Province (No. tsqnz20221171 for Y.Z), Wuhan Knowledge Innovation Special Project ‘Dawning Program’ (2023020201020352 for L.L) and the First-Class Discipline Team of 10.13039/501100003996Kunming Medical University (2024XKTDPY16).

## Declaration of competing interest

The authors declare the following financial interests/personal relationships which may be considered as potential competing interests:Ceshi Chen has patent #ZL 2025 1 0175303.5 licensed to Kunming Medical University. If there are other authors, they declare that they have no known competing financial interests or personal relationships that could have appeared to influence the work reported in this paper.

## Data Availability

Data will be made available on request.
