# Supplementary material for: Self-enriching nanozyme with photothermal-cascade amplification for tumor microenvironment-responsive synergistic therapy and enhanced photoacoustic imaging
Source: Mater Today Bio. 2025 Aug 23;34:102230. doi: 10.1016/j.mtbio.2025.102230 (PMC12415079; doi:10.1016/j.mtbio.2025.102230)
Supplement: Multimedia component 1 [file mmc1.docx]

**Self****-Enriching Nanozyme with Photothermal-Cascade Amplification for Tumor Microenvironment-Responsive Synergistic Therapy and Enhanced Photoacoustic Imaging**

Xi Zhu^1, #^, Yang Zhang^2, #^, Yufei He^3, #^, Li Li^4^, Xiaofei Luo^5^, Ran Zhao^3^, Xiaoying Yan^3^, Ceshi Chen^1,6*^

^1^Yunnan Key Laboratory of Breast Cancer Precision Medicine, Institute of Biomedical Engineering, Kunming Medical University, Kunming 650500, Yunnan, China.

^2^Department of Ultrasound, Shandong Provincial Hospital Affiliated to Shandong First Medical University, Jinan 250021, Shandong, China.

^3^Faculty of Basic Medical Sciences, Kunming Medical University, Kunming 650500, Yunnan, China.

^4^Gene Regulation and Diseases Lab, College of Life Science and Technology, College of Biomedicine and Health, Huazhong Agricultural University, Wuhan 430070, Hubei, China.

^5^Department of Mechanical and Vehicle Engineering, Changsha University of Science and Technology, Changsha, 410114, Hunan, China.

^6^Yunnan Key Laboratory of Breast Cancer Precision Medicine, Yunnan Cancer Hospital, The Third Affiliated Hospital of Kunming Medical University, Peking University Cancer Hospital, Kunming 650118, Yunnan, China.

^*^E-mail: [chenc@kmmu.edu.cn](mailto:chenc@kmmu.edu.cn)

^#^These authors contributed equally to this work.

**
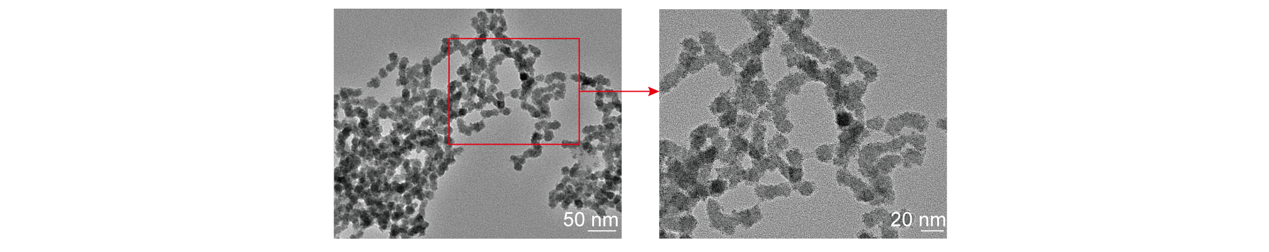
**

**Supplementary figure 1** TEM image of IrO_x_ nanoparticles synthesized without the addition of sodium citrate or pH monitoring.

**
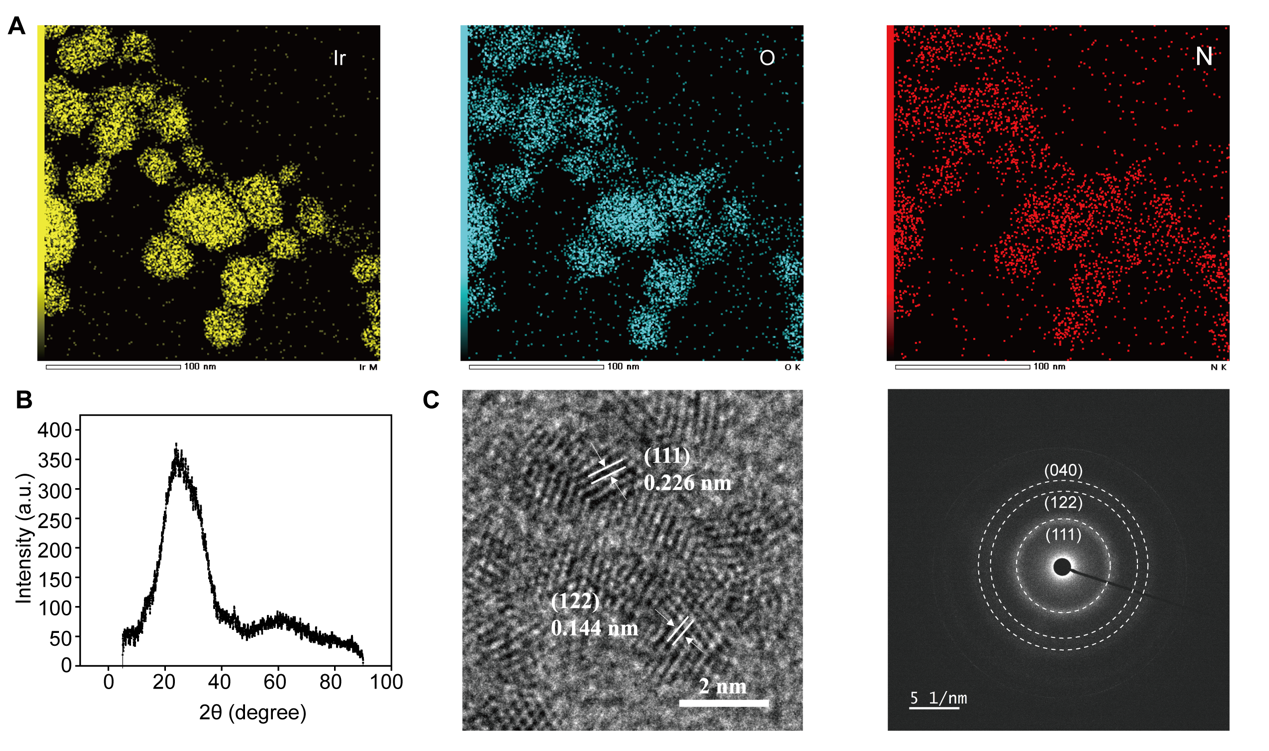
**

**Supplementary figure 2 (A)**Elemental Ir, O and N mapping images of IrO_x_-P. **(B)** XRD spectrum of IrO_x_-P particles. **(C)** HRTEM image and SAED patterns of IrO_x_-P.

**
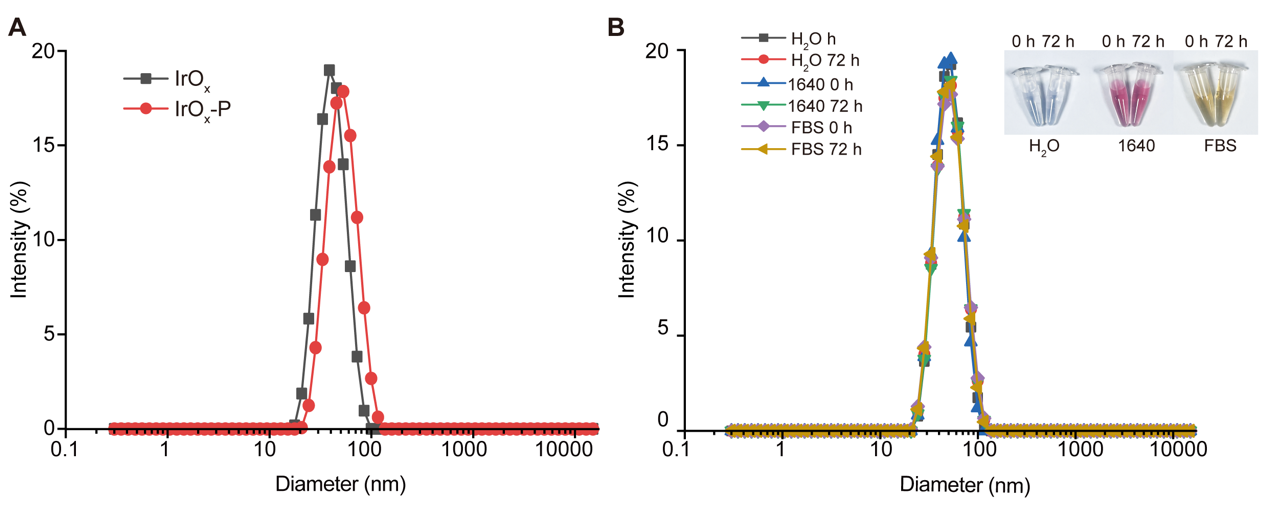
**

**Supplementary figure 3 (A)** Size distribution of IrO_x_ and IrO_x_-P nanoparticles measured by DLS. **(B)** Size distribution of IrO_x_-P nanoparticles after dissolved in H_2_O, 1640 or FBS for different timepoints.

**
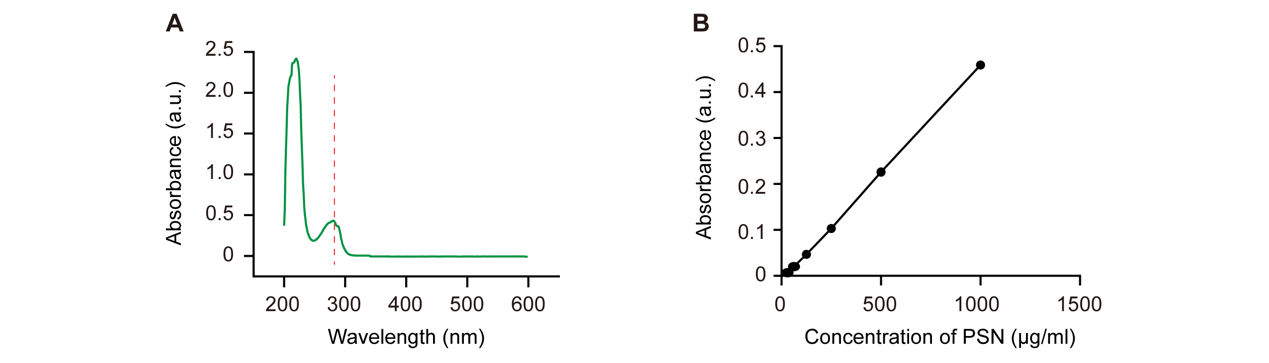
**

**Supplementary figure 4 (A)** Representative UV-Vis absorption spectrum of the PSN peptide, exhibiting a characteristic peak at approximately 220 nm attributed to peptide bond absorption, and a distinct peak near 280 nm corresponding to the specific absorbance of tryptophan (W) residues. **(B)** Standard calibration curve demonstrating the linear correlation (y = 0.0005x – 0.0102, R^2^ = 0.9998) between PSN concentration and absorbance at 280 nm.

**Supplementary Table 1** Quantitative analysis of peptide conjugation efficiency on IrO_x_ nanoparticles.

| IrO_x_  (Added) | PSN  (Added) | PSN  (Supernatant) | PSN  (Supernatant) | PSN  (Conjugated) | Conjugation efficiency |
| --- | --- | --- | --- | --- | --- |
| 50.00 mg | 5.00 mg | Absorption at 280 nm = 0.008 | 1.09 mg | 3.91 | 7.82% |

**
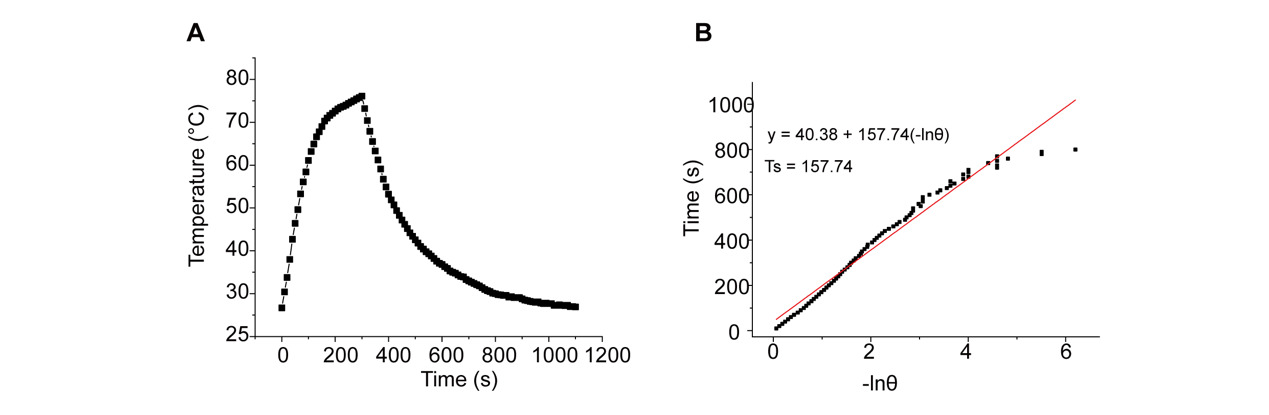
**

**Supplementary figure 5 (A)** Heating and cooling curves of IrO_x_-P nanoparticles (50 μg/ml) under 808 nm laser irradiation (1.5 W/cm^2^). **(B)** The time as a function of –lnθ obtained from the cooling cures of (A).

**Supplementary Table 2** Comprehensive summary of the physicochemical and functional parameters of IrOx-P nanoparticles.

| Particle diameter  （TEM） | Hydrodynamic size（DLS） | ς potential | Photothermal conversion efficiency | Conjugation efficiency |
| --- | --- | --- | --- | --- |
| 36.4 ± 3.7 nm | ~49.7 nm | -15.6 mV | 52.6% | 7.82% |

**
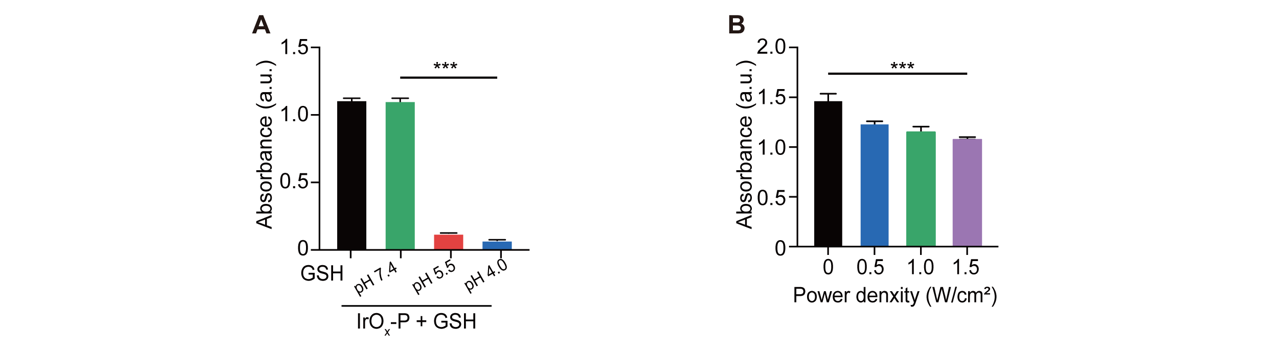
Supplementary figure 6** Quantification of GSH consumption under different pH conditions **(A)** and different laser power densities **(B)** using DTNB assay, measured by absorbance at 412 nm. Data are presented as mean ± s.d. ^*^^**^P < 0.001.

**
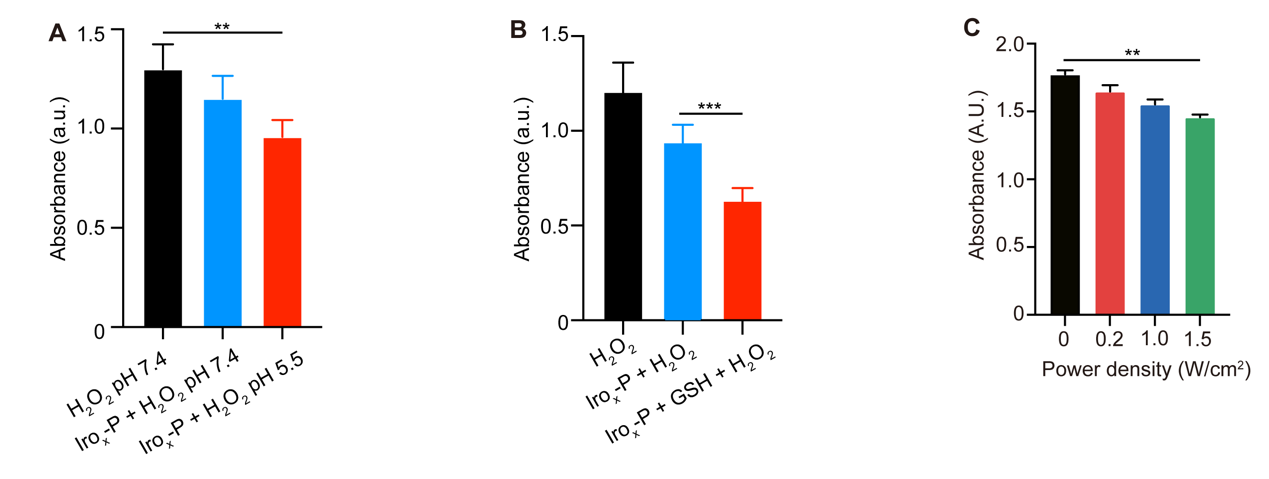
**

**Supplementary figure 7 (A)** IrO_x_-P-mediated ·OH production with different pH values assessed by MB degradation via absorbance at 660 nm. **(B)** IrO_x_-P-mediated ·OH production in the presence/absence of GSH pre-treatment via absorbance at 660 nm. **(C)** IrO_x_-P-mediated ·OH production under 808 nm laser irradiation with different power via absorbance at 660 nm. Data are presented as mean ± s.d. ^**^P < 0.01, ^***^P < 0.001.


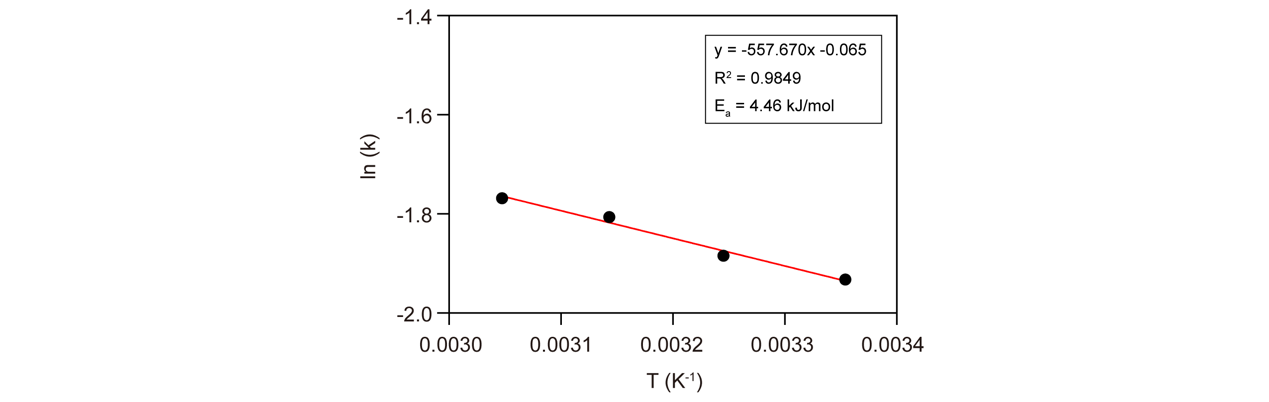


**Supplementary figure 8** Arrhenius plot for determining the activation energy (E_a_) of the temperature-enhanced POD-like reaction catalyzed by IrO_x_-P. The natural logarithm of the initial rate constants (ln [k]) was plotted against the reciprocal of absolute temperature (1/T, K⁻¹). The linear fit yields a slope of −E_a_/R (R = 8.314 J/mol⋅K^-1^).


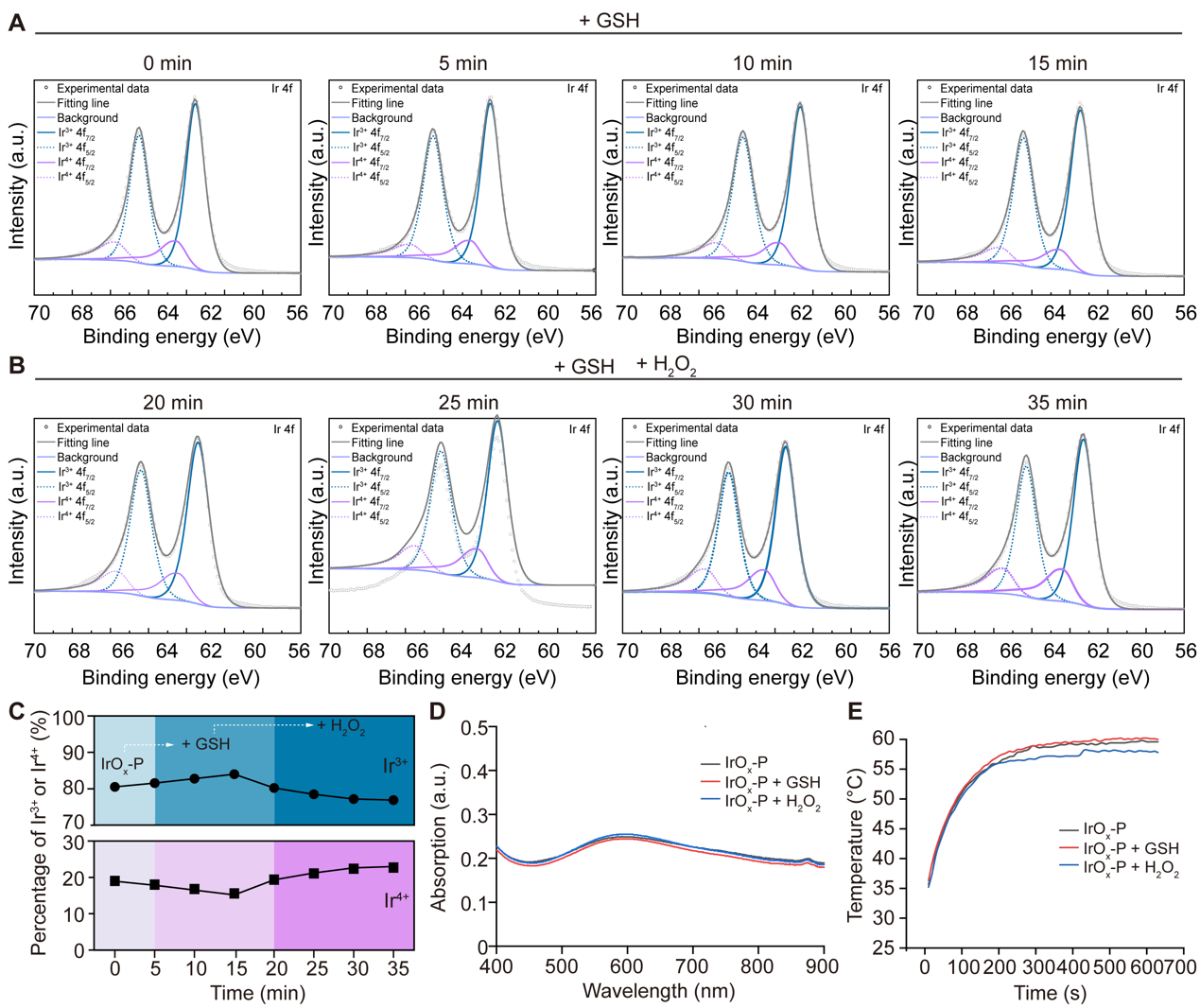


**Supplementary figure 9** Ir 4f XPS spectrum of IrO_x_-P nanoparticles (100 μg/ml) after sequential reactions with GSH (1 mM) **(A)** and H_2_O_2_ (10 mM) **(B)** at different time points. **(C)** Variations in Ir^3+^ and Ir^4+^ ratios during sequential reactions illustrated in (A) and (B). **(D)** Absorption spectra of IrO_x_-P nanoparticles after reaction with GSH or H_2_O_2_ for 30 mins. **(E)** Photothermal heating curves of IrO_x_-P nanoparticles after reaction with GSH or H_2_O_2_ for 30 mins.

**Supplementary table 3** Ir content detected by ICP-AES after reaction with GSH or H_2_O_2_ for 30 mins_._

| Groups | IrO_x_ | IrO_x_ + GSH | IrO_x_ + H_2_O_2_ |
| --- | --- | --- | --- |
| Ir content (μg/ml) | 643.6 | 634.5 | 653.4 |

**
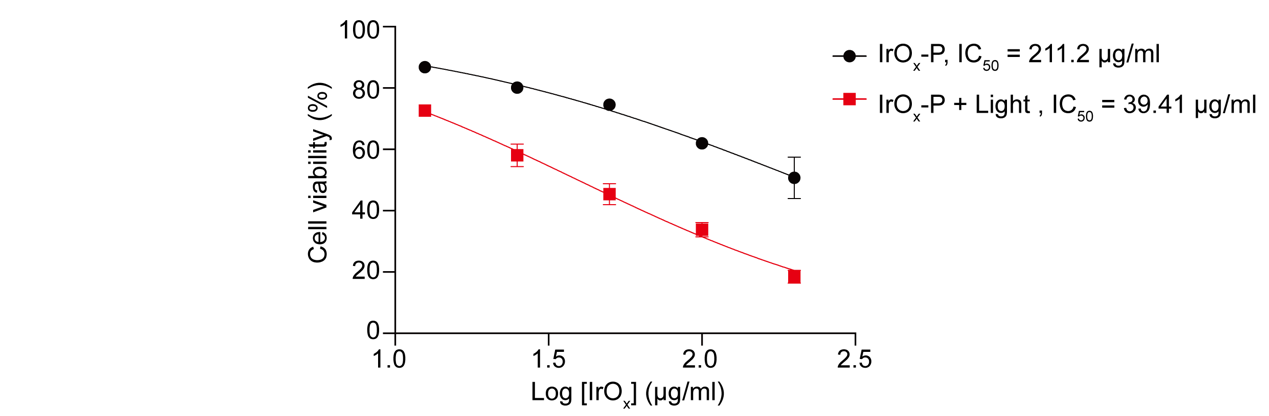
**

**Supplementary figure 10** IC_50_ values of IrO_x_-P and IrO_x_-P with light irradiation against 4T1 cells.

**
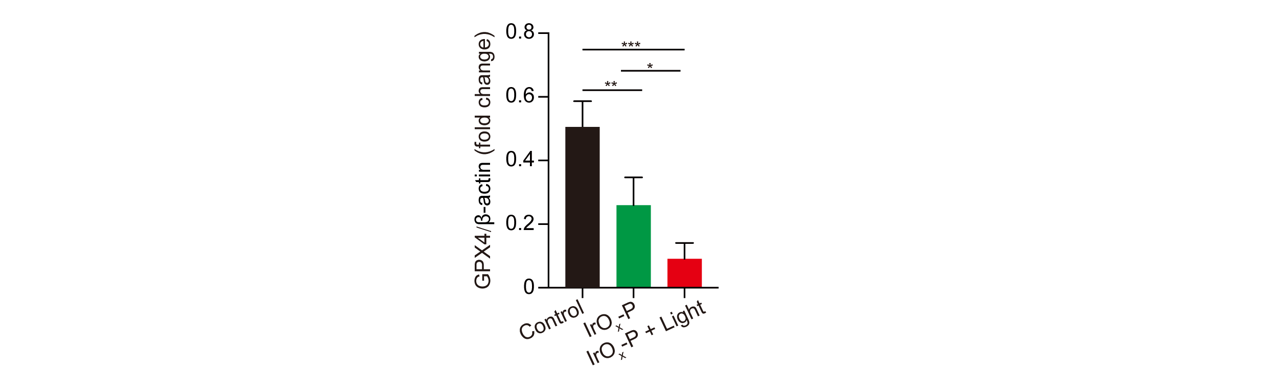
**

**Supplementary figure 11** The relative expression of GPX4 in 4T1 cells after indicated treatments. Data are presented as mean ± s.d. ^*^P < 0.05, ^***^P < 0.01, ^***^P < 0.001.

**
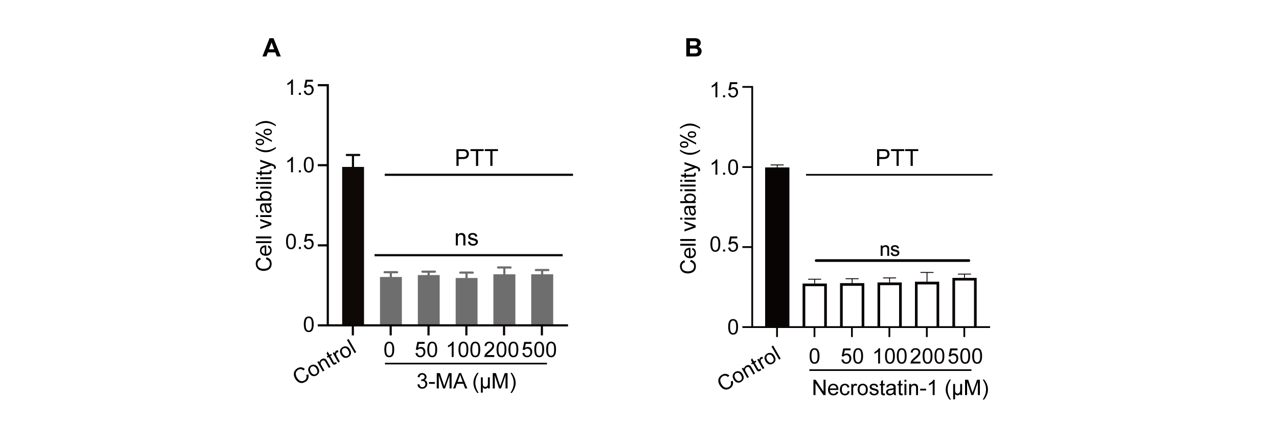
****Supplementary figure 12** Relative cell ability of PTT-treated 4T1 with the addition of 3-MA **(A)** and Necrostatin-1 **(B)**. Data are presented as mean ± s.d.
