# Supplementary material for: Self-enriching nanozyme with photothermal-cascade amplification for tumor microenvironment-responsive synergistic therapy and enhanced photoacoustic imaging
Source: Mater Today Bio. 2025 Aug 23;34:102230. doi: 10.1016/j.mtbio.2025.102230 (PMC12415079; doi:10.1016/j.mtbio.2025.102230)
Supplement: Multimedia component 2 [file mmc2.docx]

**
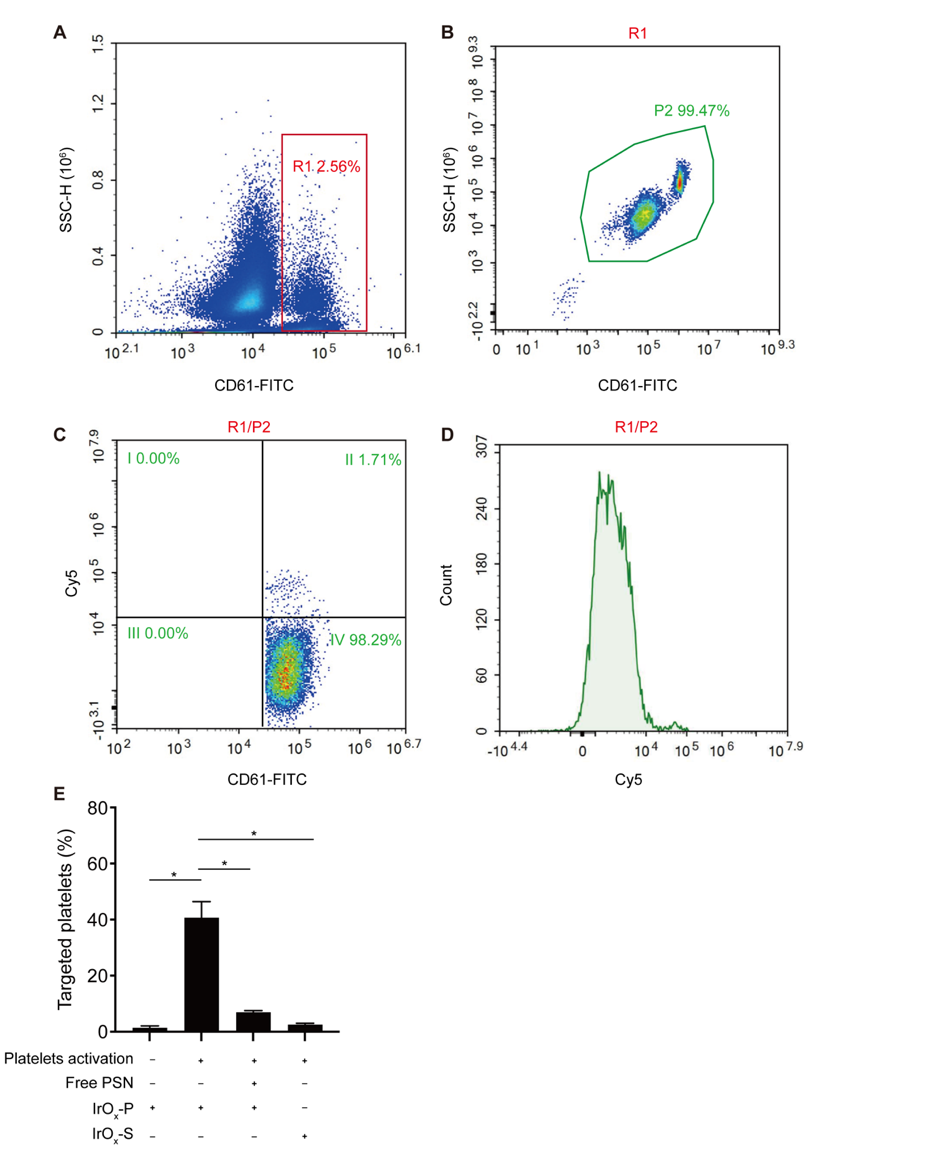
**

**Supplementary figure 13 (A-D)** Gating strategy of activated platelets. In the CD61 vs SSC dot plot (A), the CD61-positive population (R1) was selected. The R1 dot plot (B) reveals three distinct populations: (1) CD61-positive/low SSC, primarily consisting of single platelets; (2) CD61-positive/high SSC, mainly comprising blood cells with adherent platelets; and (3) CD61-positive/very low SSC, predominantly consisting of platelet-derived fragments. Based on this, the P2 gate was defined to include the single platelet population. Within the P2 gate, a CD61 vs Cy5 dot plot was used for dual-parameter analysis (C), and the double-positive results were quantified (D). **(E)** Proportion of IrO_x_-P-Cy5/ CD61 double positive platelets with different treatments. Data are presented as mean ± s.d. ^*^P < 0.05.

**
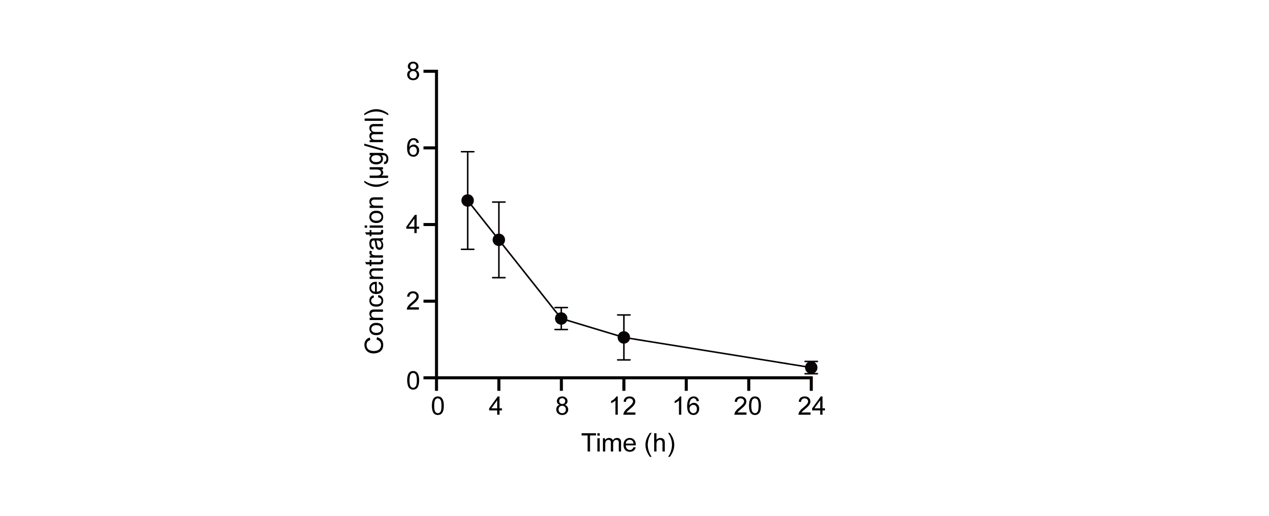
**

**Supplementary figure 14** Pharmacokinetic analysis of IrO_x_-P-Cy5 nanoparticles in blood at different time points post intravenous injection. Data are presented as mean ± s.d.

**
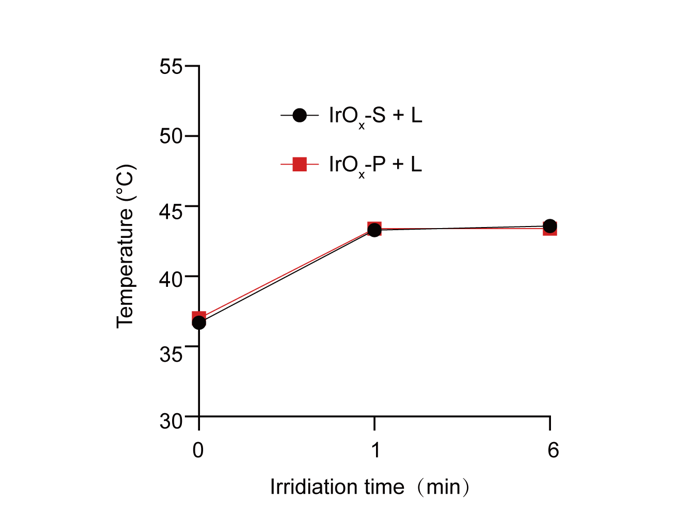
**

**Supplementary figure 15** Temperature monitoring of local tumor during first-stage light irradiation.

**
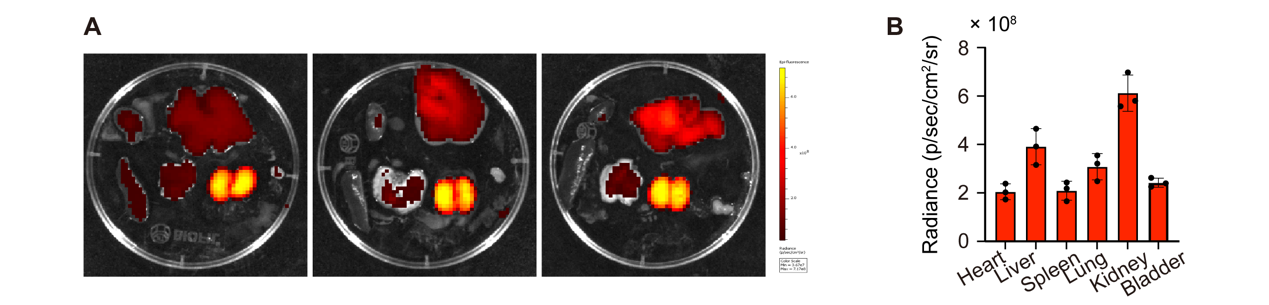
**

**Supplementary figure 16** **(A)** Ex vivo fluorescence images of major organs harvested from three mice at 12 hours post-intravenous injection of IrO_x_-P nanoparticles. Organs were arranged in the following order in culture dishes: heart, liver, spleen, lung, kidney, and bladder. **(B)** Quantitative analysis of signal intensity in each organ. Data are presented as mean ± s.d.

**
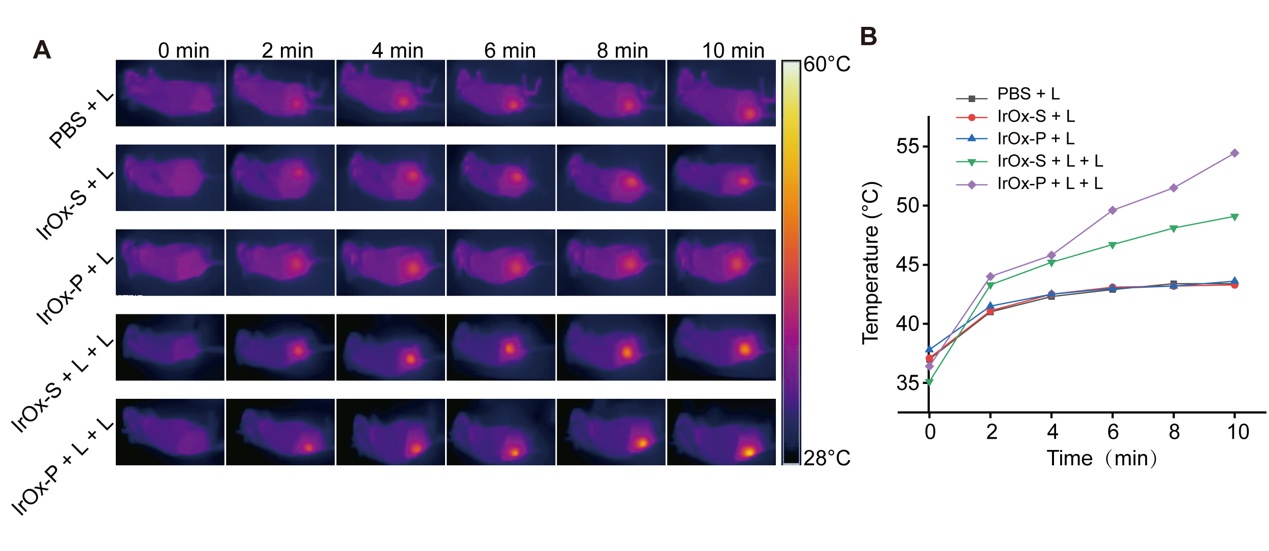
**

**Supplementary figure 17** Temperature monitoring of local tumor under first-stage or two-stage laser irradiation after intravenous injection of IrO_x_-P or IrO_x_-S.

**
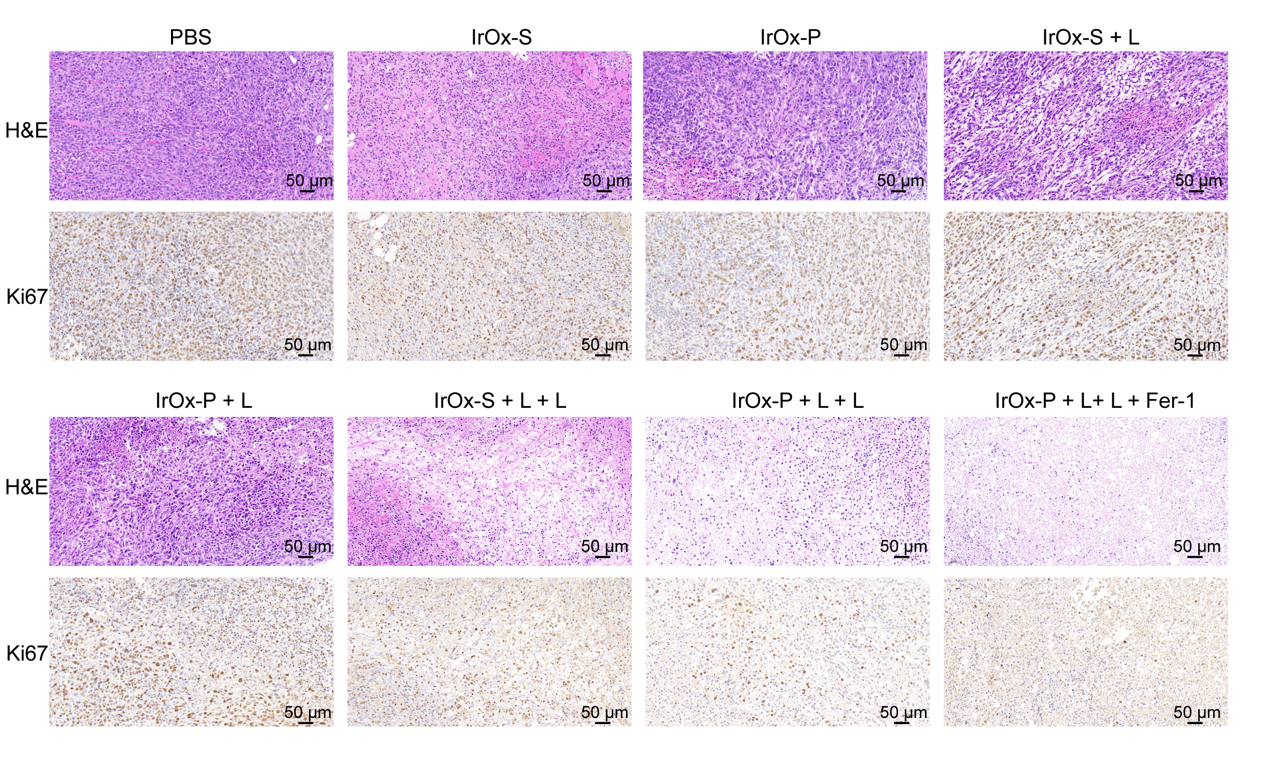
Supplementary figure 18** H&E and Ki67 staining of 4T1 tumor-bearing mice after different treatments.

**
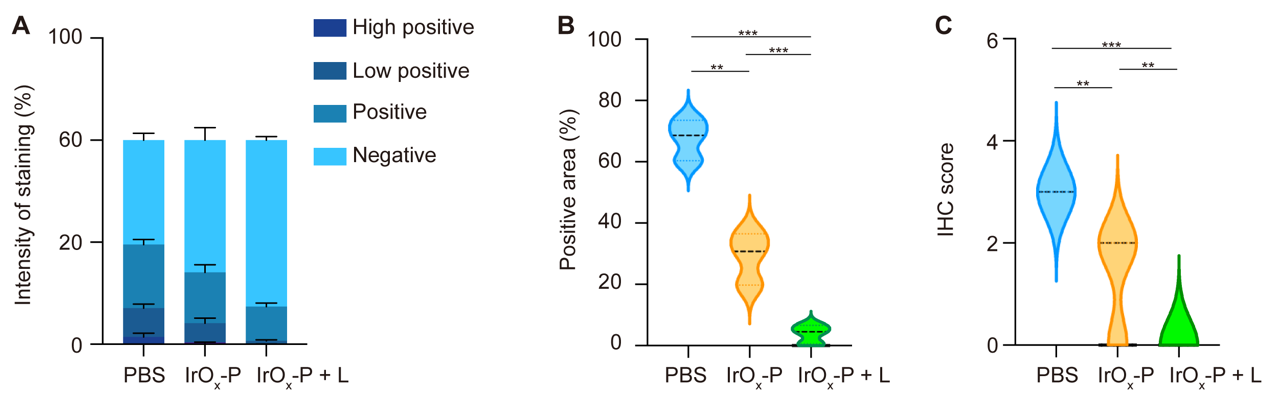
**

**Supplementary figure 19 (A)** Quantitative analysis of GPX4 immunohistochemical staining intensity in tumor tissues post-treatment. **(B)** Quantitative analysis of the GPX4-positive area in tumor tissues. **(C)** Quantitative IHC scores of GPX4 staining in tumor tissues.

**
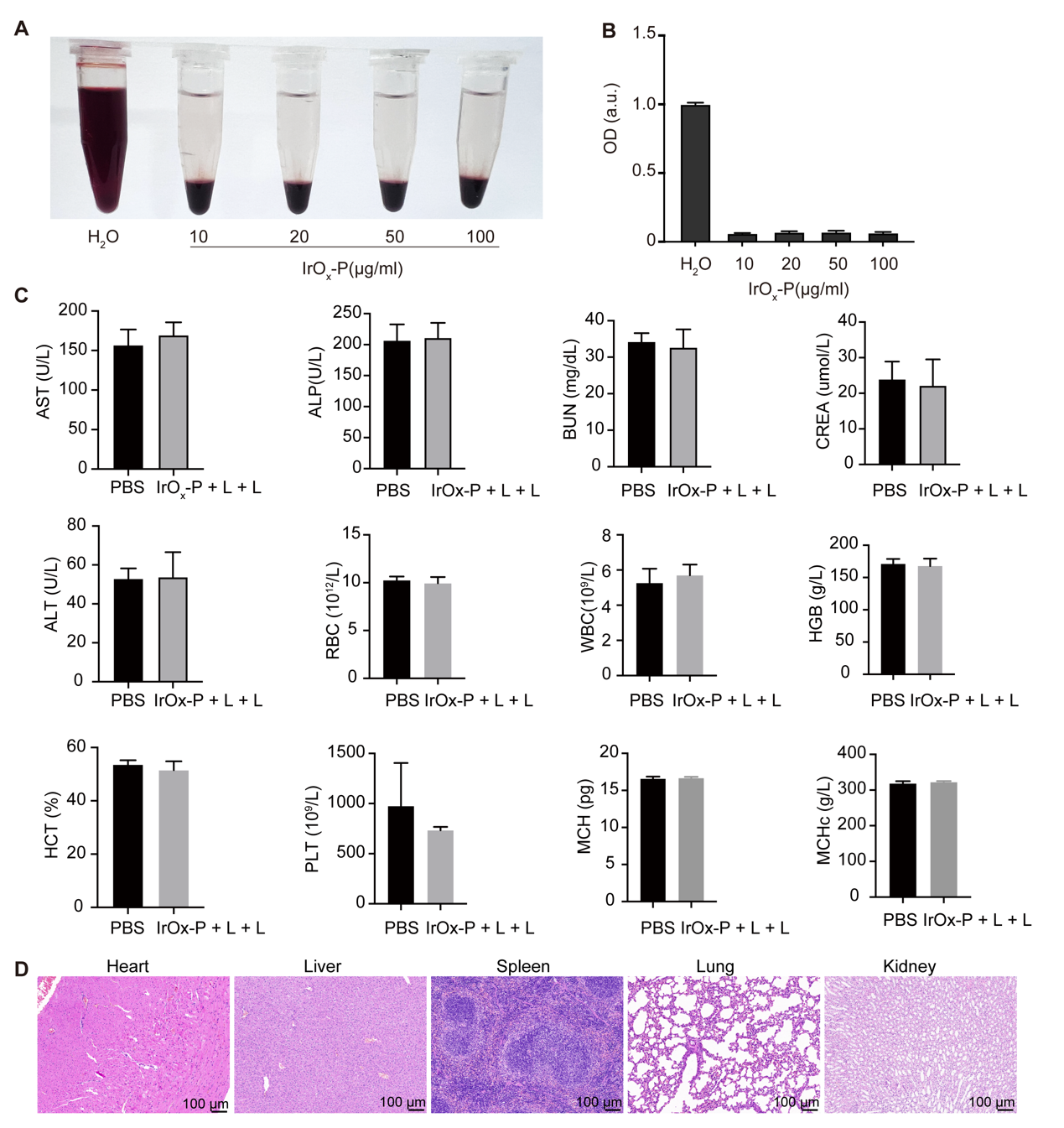
**

**Supplementary figure 20 (A)** Hemolysis test and **(B)** quantification of IrO_x_-P. **(C)** Routine blood tests ad blood biochemistry analysis at 18 days after IrO_x_-P injection and two-stage light irradiation. **(D)** H&E staining for major organ at 18 days after IrO_x_-P injection and two-stage light irradiation.


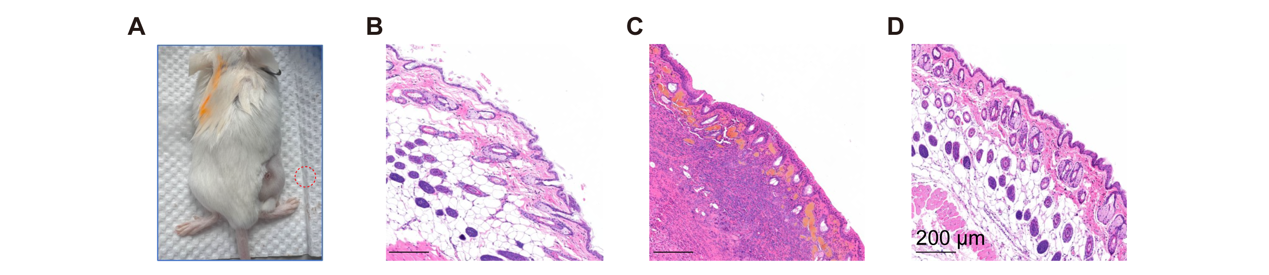


**Supplementary figure 21 (A)** Photographic images of the irradiated skin region 7 days after PTT. **(B)** H&E staining of normal skin tissue. **(C)** H&E staining of the irradiated skin region 7 days after PTT. (D) H&E staining of the irradiated skin region 18 days after PTT.

**
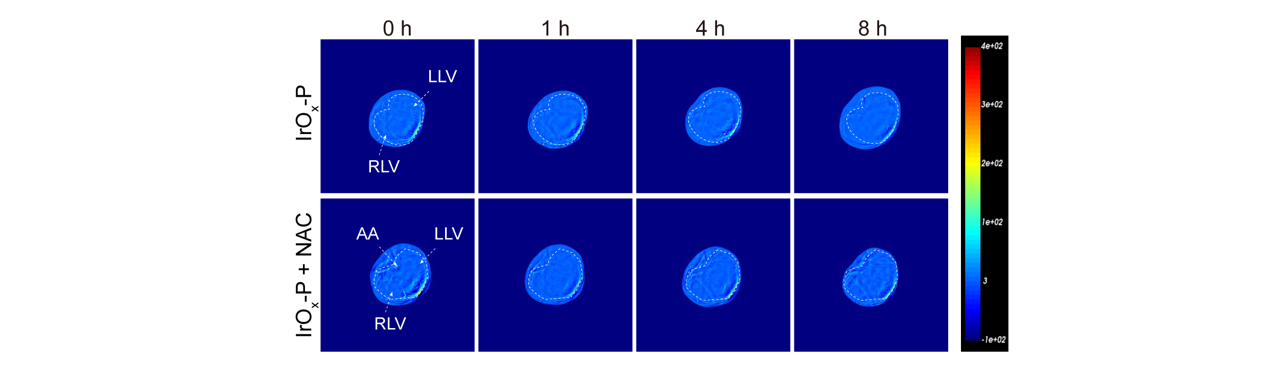
**

**Supplementary figure 22** In vivo PA imaging of liver in 4T1 tumor-bearing mice after intravenous injection of IrO_x_-P (10 mg/kg) with or without NAC pretreatment. LLV: left lobe of the live; RLV: right lobe of the liver; AA: aorta abdominalis.
